# Physicians’ Experience with COVID-19 Vaccination: A Survey Study

**DOI:** 10.3390/healthcare9121746

**Published:** 2021-12-17

**Authors:** Alina Dima, Ciprian Jurcut, Daniel Vasile Balaban, Valeriu Gheorghita, Ruxandra Jurcut, Augustin Catalin Dima, Mariana Jinga

**Affiliations:** 1 Department of Rheumatology, Colentina Clinical Hospital, 020125 Bucharest, Romania; alina_dima@outlook.com; 2 Central Military Emergency University Hospital Dr. Carol Davila, 010825 Bucharest, Romania; cjurcut@gmail.com (C.J.); valeriu.gheorghita@umfcd.ro (V.G.); dimagusti@gmail.com (A.C.D.); mariana.jinga@umfcd.ro (M.J.); 3 Faculty of Medicine, Carol Davila University of Medicine and Pharmacy, 020021 Bucharest, Romania; ruxandra.jurcut@umfcd.ro; 4 Department of Cardiology, Institute for Cardiovascular Diseases C.C. Iliescu, 022328 Bucharest, Romania

**Keywords:** vaccine hesitancy, SARS-CoV2, COVID-19, coronavirus

## Abstract

Background: COVID-19 vaccine hesitancy remains high in the general population and is the main determinant of low vaccination rates and of the fourth pandemic wave severity in Romania. Additional information is needed to raise awareness over vaccine efficiency and the safety profile. Objective: To assess self-reported experience related to COVID-19 vaccination in Romanian physicians. Design, Setting, and Participants: A cross-sectional, self-administered questionnaire-based survey, distributed online in the period 24 March to 24 May 2021. The survey included 30 cascade questions with skip logic filters. All physicians included filled in the questionnaire voluntarily and anonymously. Not all respondents filled in all questions. Main outcome and measure: Primary outcomes addressed were related to the COVID-19 vaccine safety profile. Results: 407/467 (87.15%) of the respondents’ physicians were fully vaccinated, mostly with the Pfizer—BioNTech (Comirnaty)—BNT162b2 vaccine, with the peak of immunization in January 2021, with almost four-fifths of the study participants. Regarding COVID-19, almost 20% physicians had the infection and one declared COVID-19 re-infection. A number of 48/420 (11.42%) and 47/419 (11.22%) of the vaccinated physicians did not report any side effects after the first or second vaccine dose. However, most of the side effects reported were minor. Only 50/360 (13.88%) physicians reported the vaccine side effects on the dedicated online national platform. Approximately 40% respondents checked the anti-spike SARS-CoV2 antibodies’ titer after complete vaccination, of which two cases reported indeterminate levels. Lower anti-spike SARS-CoV2 antibodies’ titer of 100–1000 times the laboratory limit was more frequent in naive physicians (36.95% versus 14.28%, *p* = 0.012), moderate titers were similar, while very high levels, more than 10,000 times laboratory limit, were more frequent in physicians with previous COVID-19 infection (2.17% versus 42.85%, *p* < 0.001). Conclusions and relevance: In this cross-sectional survey study on the COVID-19 vaccination among Romanian physicians, we describe a safety vaccination profile among Romanian physicians.

## 1. Introduction

The severe acute respiratory syndrome coronavirus 2 (SARS-CoV-2) disease (COVID-19) become shortly a public emergency and was declared pandemic by World Health Organization (WHO) on 11 March 2020.

Despite efforts, an effective curative anti-viral SARS-CoV2 treatment has not been found yet. Therefore, on move to mitigate the virus spread, preventive measures such as lockdown, safe distancing, mask wearing in public, well-ventilated spaces, the need for clean hands were promoted [1,2].

Further, to pave a way for an exit strategy, COVID-19 vaccine development became a priority worldwide. Therefore, in December 2020 the COVID-19 vaccination was started. In many countries, physicians were part of the first round of the vaccination schedule given the risk of high exposure [3,4].

SARS-CoV2 infection was reported to be more frequent in healthcare practitioners due to increased exposure [5]. One review showed that more than 20% of the healthcare workers worldwide reported COVID-19 vaccine hesitancy [6,7]. However, the reported willingness to be vaccinated is higher among healthcare workers when compared to general population [3,8]. Concerns related to COVID-19 vaccine safety and effectiveness against new variants, longevity of the immune response, related side effects, or not trusting the government were declared [6,9]. Tailored communication strategies are needed for to increase the COVID-19 vaccine-related willingness [10].

The primary aim of this research was to investigate the Romanian physicians’ experience regarding the COVID-19 vaccination.

## 2. Methods

### 2.1. Study Design and Setting

Descriptive, cross-sectional study with prospective inclusion of Romanian physicians. An online open questionnaire-based survey was conducted in accordance with currently available recommendations for E-surveys [11]. The survey consisted of 30 cascade questions on 12 pages and was initially tested by the study authors. The necessary changes were performed before starting the survey distribution and the respondents’ inclusion (Supplementary File S1).

The first two questions of the survey were designed as filter with skip logic for selecting only physicians settled in Romania as respondents. In addition, the first survey part included questions regarding the COVID-19 vaccination, while the second one covered the COVID-19 disease. Two filter questions were designed throughout the survey, with skip logic if the items in questions were not applicable for the survey, namely question Q 8 filtered the responses of the physicians that were vaccinated (Q 9–Q 23), while question 24 the physicians that had the COVID-19 diagnosis (Q 25–Q 30).

### 2.2. Participants

Eligible participants were Romanian physicians that voluntarily (no incentives were offered) agreed to took part in the survey. All physicians included filled out the survey questions during a two-month period (24 March to 24 May 2021). Responding to all questions was not mandatory. The timing of data collection was set approximately one year after the start of SARS-CoV2 infection in Romania, during the third wave of the pandemic.

### 2.3. Survey Instruments

The final survey form was introduced on a dedicated platform (SurveyMonkey^®^ Momentive Inc., San Mateo, CA, USA) and a collector link was automatically generated. The link was posted on the Facebook page of the Romanian Society of Internal Medicine and then shared in online professional Facebook groups dedicated to Romanian physicians. The questionnaire was administered only in Romanian. In order to avoid bias, the survey could have been completed only once, based on the IP address. We tried to limit the tendency for inaccurate answers by using an anonymously self-complete questionnaire ballot-box with forced-choice items and neutral questions. The responses were randomized, so the statistics interpreted only anonymous data. No personal identification information of respondents was stored during collection of data from completing the survey.

### 2.4. Ethics

After accessing the survey link, an introduction was available for respondents, explaining the premises and the terms of participation for this research. The study participation did not pose any risk. The survey filling out was completely voluntary and implied the physicians’ consent for the data analysis and presentation. Ethics approval was obtained from the local Ethics Board (No 5752/2021).

### 2.5. Statistical Analysis

Descriptive statistics are reported from the data gathered through the questionnaire (see Supplementary File S2). Sample size of 382 measurements was calculated for 95% confidence interval and 5% margin of errors. Statistics were completed using the SPSS Microsoft Excel and SPSS Statistics v25 software Armonk, NY, USA.

## 3. Results

### 3.1. General Data

The survey consisted in 30 questions arranged on 6 pages. The completion rate was 93%, the completeness rate of 92%, with typical time spent for survey fill in of 4 minutes.

Altogether 509 respondents started the survey, of which 20/509 (3.93%) respondents were excluded for not being physicians and then another 11/488 (2.25%) respondents for not being settled in Romania. Not all physicians that started the survey completed all questions, response rate 81.53% (Supplementary File S2).

The survey included in majority respondents of female gender 416/467 (89.08%), with ages between 20–29 years (20.77%), 30–39 years (25.70%), 40–49 years (25.05%), 50–59 years (22.27%) from various medical specialties in different stages of professional career (see Supplementary File S3—Supplementary Table S1).

In our study group, 407/467 (87.15%) respondents were fully vaccinated. Pfizer—BioNTech (Comirnaty)—BNT162b2 vaccine, both first dose and complete immunization, was most frequently used (413/420 physicians, 98.33%). The vaccination started in December 2020 and had highest rates in January 2021, when 332/420 (79.05%) respondents had the first vaccine dose (Table 1).

### 3.2. Side Effects Reported after COVID-19 Vaccination

Regarding the COVID-19 vaccine, only 48/420 (11.42%) (Supplementary Table S2) and 47/419 (11.22%) (Table 2) of the recipients did not report any side effects after the first or second dose. However, most of the side effects were minor, resolved spontaneously in 152/417 (36.45%) cases.

### 3.3. The Impact of the COVID-19 Vaccination

Among the respondents, 310/360 (86.11%) of the ones that responded to have post-vaccinal adverse effects did not report them on the dedicated national platform. Regarding severity, 176/421 (41.81%) of the participants answered that sick leave for the side effects secondary to COVID-19 vaccination is not needed, while 203/421 (48.22%) considered that a sick leave might be very rare needed (see Table 3).

The main reason for not being vaccinated was recent COVID-19 disease 8/24 (33.33%). Further, 4/24 (16.67%) declared history of allergy or anaphylactic reaction. However, 5/24 (20.83%) had doubts over vaccine efficacy.

### 3.4. The Experience of the COVID-19 Disease

In total, 72/421 (17.10%) of the physicians had COVID-19 and one (0.24%) declared to have been infected twice with SARS-CoV2. High rates of SARS-CoV2 infection among doctors were registered in the last three months of 2020, accounting for 46/73 (63.01%) of cases (Supplementary Table S3). None of the physicians included received the SARS-CoV2 infection after vaccination (follow-up for less than 6 months after complete vaccination).

Regarding severity, most of the COVID-19 cases were declared as mild 49/73 (67.12%) and only one as severe disease (1.37%). Further, 12/73 (16.43%) COVID-19 cases needed hospitalization. (Supplementary Table S4). Paracetamol and vitamins were the most used drugs (Supplementary Table S4).

### 3.5. Serological Response to the COVID-19 Vaccination

In total, 159/419 (37.94%) determined the anti-spike SARS-CoV2 antibodies’ titer after complete vaccination, of which two (1.25%) cases had indeterminate levels (in both cases, determination was conducted within 1–2 weeks after vaccination in physicians with no history of infection).

When compared to physicians that had COVID-19 before vaccination, the proportion of uninfected vaccinated physicians with anti-spike SARS-CoV2 antibodies’ titer between 1000–5000 or 5000–1000 times the laboratory limit was similar, 36/138 (26.08%) versus 4/21 (19.04%), *p* = 0.201 and 6/138 (4.34%) versus 2/21 (9.52%), *p* = 0.559. However, lower titers of 100–1000 times the laboratory limit were more frequent in SARS-CoV2 naive physicians, 51/138 (36.95%) versus 3/21 (14.28%), *p* = 0.012, while higher than 10,000 times the laboratory limit were more frequent in the physicians previously exposed to SARS-CoV2, 3/138 (2.17%) versus 9/21 (42.85%), *p* < 0.001.

## 4. Discussion

The survey was released about one year after the first COVID-19 cases in Romania [12], during the period when lockdown measures were in force according to the State of Alert on the Romanian territory during the third COVID-19 wave [13].

As in other regions [14], the physicians were included among the first vaccinated subjects. The rate of vaccinated physicians is higher than that of the Romanian general population, only 10.27% on 1 May 2021 and 27.4% on 1 October 2021 [15]. Higher vaccination rates for health professionals were reported in other countries also [16,17]. “To Protect Myself, My Friends, Family, Workmates and Patients” were main wishes declared by the physicians that received the vaccine [9]. In addition, another important point is that the healthcare professionals have ethical, legal, and moral obligations to protect their patients [8]. In one research, the main reasons for not being vaccinated were a pending vaccination appointment and safety concerns [4]. Moreover, frequent social media exposure and interpersonal discussion potentially might increase the vaccination willingness [18]. More data and information on the safety and efficacy should be transparently provided within tailored communication strategies on COVID-19 vaccine [10].

Even if the vaccination rates are higher among healthcare workers when compared to general population [4,8], the majority is against mandatory vaccination [3]. The rates of vaccine acceptance increased with age, being highest in the over-55 group [3,6,7] and also with the fear of COVID-19 [7].

One large survey that examined the levels and predictors of acceptance of an approved COVID-19 vaccine in eight Western democracies showed that the levels of vaccine acceptance fall below estimates of the required for obtaining herd immunity [19]. These results drew attention on the long-term importance of building trust in preparations for health emergencies such as the current pandemic [19].

It was shown that the acceptance rates were larger for mRNA than for vector-based (90% versus 50%) vaccines [20]. In addition, the acceptance rate is higher in physicians when compared to nurses or other healthcare workers [6,7,21,22]. Even if frequent interpersonal interactions and discussion over vaccination process are positively associated with intention of COVID-19 vaccination, the discussion with laymen had larger effect than those with medical professionals [18].

Overall, the COVID-19 vaccine hesitancy was significantly linked to the embrace of vaccine conspiracy beliefs [22]. Regarding this, it is important to note that targeting the relevant information appropriately should be a key point for the success of vaccination programs [5].

Due to vaccine hesitancy, many vaccine doses remained unused in Romania and were subsequently send to other countries. The low vaccination rates were also the main cause of the severity of the fourth COVID-19 pandemic wave in Romania that started in September 2021 and when the highest daily mortality rates secondary to COVID-19 were registered. Contrary to other European countries in periods when vaccines were not available, the overload of the medical system in Romania would have been preventable by through effective control measures of fake news regarding vaccines and a good campaign of information over vaccine efficiency and safety profile. We want so to raise awareness here over the safety profile of the COVID-19 vaccination in Romania through the experience of the Romanian physicians’ vaccination.

At beginning, there were many discussions regarding a fair vaccination prioritization [8,23]. The healthcare workers were included in Romania in the first phase of the vaccination campaign, being considered a high-risk population, such as those persons with comorbidities or those older than 65 years.

Regarding vaccine efficacy, this was reported for the initial strains to be as high as 95% for COVID-19 mRNA vaccine BNT162b2 (Pfizer), 94.1% for mRNA-1273 vaccine (Moderna), and 70.4% for ChAdOx1 nCoV-19 vaccine/AZD1222 (AstraZeneca), but dropped for the new strains (delta) [24]. The lack of COVID-19 immune response might be due to lack of natural efficient immunity or to high specificity of the SARS-CoV2-2 genomic mutations [25]. In our research, the anti-spike SARS-CoV2 antibodies were negative in only 1.25% of the vaccinated physicians, but event in these cases, both determinations were performed in less than 14 days in these cases and might be inconclusive.

The infection rate was reported to decrease from 1.3/10,000 person-days in naive to 0.30 for fully vaccinated healthcare workers [26]. For hospital employees, the main concerns listed to refuse vaccination were related to vaccine safety and side effects (49.4%), followed by distrust in the vaccine’s efficacy (41.1%) [27]. Most cases of physicians who have had COVID-19 (63.01%) were declared in the last three months of 2020, during the second wave, the most aggressive part of pandemic in Romania until the fourth wave in September 2021 and before having available a COVID-19 vaccine.

The SARS-CoV-2 vaccines were shown to have high immunogenicity. Thereby, 99.8% of all vaccinated subjects showed seropositivity to anti-SARS-CoV-2 IgG and also, more than 80% had IgG concentrations > 200 AU/mL [28]. In our survey, very high anti-spike SARS-CoV2 antibodies titers (more than 10,000 times laboratory limit) were more frequent in physicians with post-infection immunity prior to vaccination. It was so previously questioned if a single vaccine dose might be sufficient to induce effective response [29]. Even if the determination of the anti-spike SARS-CoV2 was not standardized yet and a cut-off for protective level was not defined, there are data that suggest that the anti-spike SARS-CoV2 antibodies levels are correlated with the protection conferred [30,31]. However, having a vaccine booster is not related to the anti-spike SARS-CoV2 antibodies levels. In addition, the anti-spike SARS-CoV2 antibodies determination is not routinely recommended. Ongoing studies are searching for the homologous and heterologous booster vaccination tolerance and immunogenicity in adults with complete primary COVID-19 vaccination [32].

### Limitations

The current study has some limitations. The strictly online distribution was one limitation. Furthermore, the survey was open, and we also could not describe the non-responders. However, the target population were physicians and skip logic questions were designed for responders’ selection. Moreover, methods to avoid bias were used: the questionnaire was self-administered, self-completely anonymously (ballot-box method), using neutral and adaptive questions with non-response options provided. Not least, here is a limit in using an unvalidated questionnaire, but this was balanced by the thorough literature search performed by the study authors in developing the survey items. Another limitation is that this research does not offer a comparation with the general population.

## 5. Conclusions

COVID-19 vaccination proved to have a good safety profile among physicians. None of the COVID-19 cases was registered with 6 months after full vaccination. If moderate titers were similar, very high anti-spike SARS-CoV2 antibodies were found mostly in physicians with prior COVID-19 infection before vaccination.

### Strengths and Limitations of This Study

- The research presents data on vaccine efficiency and safety in one of the European countries with lowest rate of vaccination.
- It is important to note that none of the COVID-19 cases was declared after full vaccination and within 6 months after vaccination.
- Very high anti-spike SARS-CoV2 IgG antibodies titers were more frequently observed in vaccinated physicians who previously obtained natural immunity.
- The online questionnaire distribution is the one limitation of this research.

## Figures and Tables

**Table 1:** General data regarding the physicians’ COVID-19 vaccination.

	Respondents, n (%)
COVID19 vaccination (Q8)	
Yes, both doses	407/467 (87.15%)
Yes, one dose	33/467 (7.07%)
No	27/467 (5.78%)
Reasons for not being vaccinated (Q9)	
History of allergies	3/24 (12.50%)
History of anaphylactic reaction	1/24 (4.17%)
Recent SARS-CoV2 infection	8/24 (33.33%)
Do not trust the vaccine efficiency	5/24 (20.83%)
Something else	9/24 (37.5%)
Type of COVID19 vaccine received (Q10)	
Pfizer—BioNTech (Comirnaty)—BNT162b2	413/420 (98.33%)
Moderna—mRNA-1273	3/420 (0.71%)
AstraZeneca/Oxford—AZD1222	4/420 (0.95%)
Period for the COVID19 vaccination (Q11)	
December 2020	25/420 (5.95%)
January 2021	332/420 (79.05%)
February 2021	38/420 (9.05%)
Marth 2021	24/420 (5.71%)
April 2021	1/420 (0.24%)

**Table 2:** Side effects reported after COVID-19 vaccination.

	Respondents, n (%)
Side effects after the second vaccine dose (Q16)	
I did not receive yet the second vaccine dose	29/419 (6.92%)
None	47/419 (11.22%)
Local cutaneous changes at injection side	15/419 (3.58%)
Pain at injection site	282/419 (67.30%)
Easy-moderate allergic reaction	3/419 (0.72%)
Severe allergic reaction	0/419 (0.00%)
Cutaneous eruptions	2/419 (0.48%)
Important asthenia	99/419 (23.63%)
Sleepiness	97/419 (23.15%)
Insomnia	26/419 (6.21%)
Feverish	54/419 (12.89%)
Fever	37/419 (8.83%)
Shiver	89/419 (21.24%)
Myalgia	109/419 (26.01%)
Appetit loss	10/419 (2.39%)
Nausea/vomiting	24/419 (5.73%)
Diarrhea	3/419 (0.72%)
Headache	96/419 (22.91%)
Drowsiness	28/419 (6.68%)
Tinnitus	1/419 (0.24%)
Vertigo	13/419 (3.10%)
Odynophagia	4/419 (0.95%)
Cough	1/419 (0.24%)
Palpitations	15/419 (3.58%)
Increased value of arterial tension	6/419 (1.43%)
Adenopathy axillar/supraclavicular	36/419 (8.59%)
Something else (were completed: hypothermia, trigemini neuralgia, Raynaud phenomenon, arthralgia, trouble of concentration, zona zoster)	26/419 (6.21%)
The period after the second vaccine dose in which the side effects appeared (Q17)	
I did not receive yet the second vaccine dose	28/415 (6.75%)
Does not apply, I did not have side effects	46/415 (11.08%)
Less than 24 hours	159/415 (38.31%)
1–3 days	149/415 (35.90%)
3–7 days	19/415 (4.58%)
1–2 weeks	4/415 (0.96%)
2–4 weeks	4/415 (0.96%)
1–2 months	4/415 (0.96%)
More than 2 months	0/415 (0.00%)
Something else	2/415 (0.48%)
Treatment for the COVID-19 vaccine’s side effects (Q19)	
I did not receive yet the second vaccine dose	28/417 (6.71%)
Does not apply, I did not have side effects	50/417 (11.99%)
Nothing, spontaneous improvement	152/417 (36.45%)
Antihistamines	8/417 (1.92%)
Paracetamol	147/417 (35.25%)
Nonsteroidal anti-inflammatory drugs	75/417 (17.99%)
Corticosteroids	1/417 (0.24%)
Yes, something else	15/417 (3.60%)

**Table 3:** Impact of the COVID-19 vaccination.

	Respondents, n (%)
The COVID-19 side effect reported on the national dedicated platform (Q20)	
Does not apply, I did not have side effects	61/421 (14.49%)
Yes, I did report the side effects	50/421 (11.88%)
No, I did not report the side effects	310/421 (73.63%)
The necessity of medical sick leave for the side effects secondary to COVID-19 vaccination (Q21)	
No, there are only mild side effects	176/421 (41.81%)
Yes, very rare sick leave might be needed	203/421 (48.22%)
Yes, quite frequent sick leave is needed	37/421 (8.79%)
Yes, sick leave after vaccination is usually needed	5/421 (1.19%)
Anti-Spike SARS-CoV2 antibodies’ titer after complete vaccination (Q22)	
Does not apply, I did not make the determination	259/419 (61.81%)
Negative titer, under the laboratory titer	2/419 (0.48%)
Positive titer, 1–100 times the laboratory limit	44/419 (10.50%)
Positive titer, 100–1000 times the laboratory limit	54/419 (12.89%)
Positive titer, 1000–5000 times the laboratory limit	40/419 (9.55%)
Positive titer, 5000–10,000 times the laboratory limit	8/419 (1.91%)
Positive titer, more than 10,000 times the laboratory limit	12/419 (2.86%)
Moment after vaccination for the anti-Spike SARS-CoV2 antibodies’ determination (Q23)	
Does not apply, I did not make the determination	259/418 (61.96%)
Less than 2 weeks after the second vaccination dose	46/418 (11.00%)
2–4 weeks after the second vaccination dose	89/418 (21.29%)
1–2 months after the second vaccination dose	23/418 (5.50%)
2–3 months after the second vaccination dose	1/418 (0.24%)

## Data Availability

All available data are presented in the manuscript and the Supplementary Files.

## Supplementary Materials

The following are available online at https://www.mdpi.com/article/10.3390/healthcare9121746/s1, Supplementary File S1: the questionnaire—file from SurveyMonkey; Supplementary File S2: synthesis of results—file from SurveyMonkey; Supplementary File S3: Supplementary Table S1. General characteristics of the physicians ‘respondents in the study; Supplementary Table S2. Side effects related to the first COVID-19 vaccine dose; Supplementary Table S3. Diagnostic of the COVID-19 disease in the physicians’ respondents to the survey and Supplementary Table S4. COVID-19’s characteristics in the physicians’ respondents to the survey.
