# Supplementary material for: Physicians’ Experience with COVID-19 Vaccination: A Survey Study
Source: Healthcare (Basel). 2021 Dec 17;9(12):1746. doi: 10.3390/healthcare9121746 (PMC8702136; doi:10.3390/healthcare9121746)
Supplement: Supplementary file 1 [file healthcare-09-01746-s001.zip › Supplementary File S1.pdf]

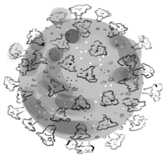

Experiența vaccinării COVID-19 pentru medicii din România

Dragi colegi medici,

Vă mulțumim că ați acceptat invitația noastră de a participa la acest sondaj online dedicat medicilor din România, care evaluează experiența privind vaccinarea COVID-19. Prin acest scurt chestionar online, invităm colegi români din întreaga țară să-și împărtășească anonim experienta vaccinării anti-COVID-19.

Sondajul include 30 întrebări cu o singură alegere sau cu alegere multiplă și completarea lui durează în jur de 5-7 minute.

Participarea la acest sondaj implică acordul de a utiliza răspunsurile pentru a se realiza o analiză a experienței actuale privind vaccinarea COVID-19 în România.

Datele colectate prin intermediul acestui sondaj vor fi procesate sub anonimat. Prin completarea acestui chestionar, vă exprimați acordul de a participa voluntar la această cercetare bazată pe întrebări.

Puteți refuza să participați la studiu sau să vă retrageți oricând înainte de a termina sondajul. Rezultatele vor fi prezentate în lucrări cu caracter stiințific, disponibile inclusiv online.

Cercetarea este derulată de un grup multidisciplinar de medici din UMF Carol Davila, București.

Pentru întrebări, vă rugăm să contactați physician.survey.covid19@gmail.com.

* 1. Sunteti medic?

- ◯ Da
- ◯ Nu

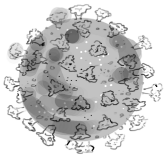

Experiența vaccinării COVID-19 pentru medicii din România

* 2. Lucrati in Romania?

- Da
- Nu

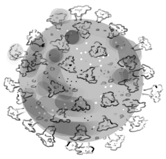

Experiența vaccinării COVID-19 pentru medicii din România

* 3. Varsta dumneavostra este:

- 20 – 29 ani
- 30 – 39 ani
- 40 – 49 ani
- 50 – 59 ani
- 60 – 69 ani
- 70 – 79 ani

* 4. Care este sexul dumneavostra?

- Masculin
- Feminin

* 5. Care este specialitatea in care activati?

- Anestezie si Terapie Intesiva
- Boli Infectioase
- Cardiologie
- Dermatologie
- Chirurgie generala
- Chirurgie toracica
- Chirurgie vasculara
- Chirurgie cardiaca
- Epidemiologie
- Gastroenterologie
- Hematologie
- Medicina de Familie
- Medicina de Urgenta
- Alteva (va rugam mentionati)
- Medicina interna
- Nefrologie
- Neurologie
- Neurochirurgie
- Oftalmologie
- ORL
- Ortopedie/Trauma
- Pediatrie
- Pneumologie
- Reumatologie
- Radiologie si Imagistica Medicala
- Urologie

* 6. Care este nivelul dumneavoastra de experienta?

- Medic rezident
- Medic specialist
- Medic primar
- Sef de sectie/Profesor
- Medic pensionar

* 7. Va rugam sa precizati cu care dintre urmatoarele comorbiditati ati fost diagnosticat/a: (raspuns multiplu posibil)

- Nu am patologii semnificative diagnosticate
- Hipertensiune arteriala
- Insuficienta cardiaca
- Insuficienta respiratorie
- Boala renala cronica fara dializa
- Dializa renala
- Altceva (va rugam precizati)
- Diabet zaharat
- Boala hepatica cronica
- Boala autoimuna
- Neoplazie
- Obezitate
- Alergii

* 8. Vi s-a administrat vaccinul anti-COVID-19?

- ◯ Da, ambele doze
- ◯ Da, doar prima doza
- ◯ Nu

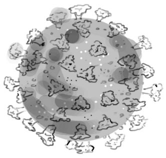

Experiența vaccinării COVID-19 pentru medicii din România

* 9. Daca NU v-ati vaccinat impotriva COVID-19, care este motivul?

- ☐ Istoric de alergii
- ☐ Istoric de soc anafilactic
- ☐ Am avut COVID-19 recent
- ☐ Nu cred in utilitatea vaccinului
- ☐ Altceva (va rugam precizati)

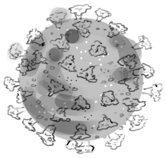

Experiența vaccinării COVID-19 pentru medicii din România

* 10. Cu ce vaccin anti-COVID-19 ati fost vaccinat/a?

- ◯ Pfizer - BioNTech (Comirnaty) - BNT162b2
- ◯ Moderna – mRNA-1273
- ◯ AstraZeneca/ Oxford – AZD1222
- ◯ Altceva (va rugam precizati)

* 11. Cand ati fost vaccinat/a impotriva COVID-19 (prima doza)?

- ○ Decembrie 2020
- ○ Ianuarie 2021
- ○ Februarie 2021
- ○ Martie 2021
- ○ Aprilie 2021

* 12. Inainte de administrarea vaccinului anti-COVID-19 +/- inaintea aparitiei oricarei reactii adverse, ati luat vreun tratament pentru a preveni eventualele reactii adverse secundare vaccinarii? (raspuns multiplu posibil)

- ☐ Nu, nu am luat niciun medicament
- ☐ Da, antihistaminic
- ☐ Da, paracetamol
- ☐ Da, AINS
- ☐ Da, altceva (va rugam precizati)

* 13. Dupa administrarea primei doze a vaccinului anti-COVID-19 ce reactii adverse ati prezentat?

(raspuns multiplu posibil)

- ☐ Nu am avut nicio reactie adversa
- ☐ Modificari cutanate la locul de injectare
- ☐ Durere la locul de injectare
- ☐ Reactie alergica usoara-moderata
- ☐ Reactie alergica severa
- ☐ Eruptii cutanate
- ☐ Astenie marcata
- ☐ Somnolenta
- ☐ Insomnie
- ☐ Subfebrilitate
- ☐ Sindrom febril
- ☐ Frison
- ☐ Mialgii
- ☐ Altceva (va rugam precizati)
- ☐ Inapetenta
- ☐ Greata/ varsaturi
- ☐ Scaune diareice
- ☐ Cefalee
- ☐ Ameteli
- ☐ Tinitus
- ☐ Vertij
- ☐ Odinofagie
- ☐ Tuse
- ☐ Palpitatii
- ☐ Cresterea tensiunii arteriale
- ☐ Adenopatie axilara/supraclaviculara

* 14. La ce interval dupa prima adminstrare a vaccinului anti-COVID-19 au aparut reactiile adverse atribuite acestuia?

- Nu se aplica, nu am avut reactii adverse
- Mai putin de 24 ore
- 1-2 zile
- 2-4 zile
- 4-7 zile
- 1-2 saptamani
- Altceva (va rugam precizati)

* 15. Ce medicamente ati luat ca tratament pentru a trata reactiile adverse aparute dupa prima administrare a vaccinului anti-COVID-19? (raspuns multiplu posibil)

- [ ] Nu se aplica, nu am avut reactii adverse
- [ ] Nimic, s-au ameliorat spontan
- [ ] Antihistaminice
- [ ] Paracetamol
- [ ] AINS
- [ ] Corticosteroizi
- [ ] Altceva (va rugam precizati)

* 16. Dupa administrarea celei de a 2-a doze a vaccinului anti-COVID-19 ce reactii adverse ati prezentat?

(raspuns multiplu posibil)

- [ ] Nu se aplica, nu am primit inca a doua doza a vaccinului
- [ ] Nu am avut nicio reactie adversa
- [ ] Modificari cutanate la locul de injectare
- [ ] Durere la locul de injectare
- [ ] Reactie alergica usoara-moderata
- [ ] Reactie alergica severa
- [ ] Eruptii cutanate
- [ ] Astenie marcata
- [ ] Somnolenta
- [ ] Insomnie
- [ ] Subfebrilitate
- [ ] Sindrom febril
- [ ] Frison
- [ ] Altceva (va rugam precizati)
- [ ] Mialgii
- [ ] Inapetenta
- [ ] Greata/varsaturi
- [ ] Scaune diareice
- [ ] Cefalee
- [ ] Ameteli
- [ ] Tinitus
- [ ] Vertij
- [ ] Odinofagie
- [ ] Tuse
- [ ] Palpitatii
- [ ] Cresterea tensiunii arteriale
- [ ] Adenopatie axilara/ supraclaviculara

* 17. La ce interval dupa cea de a 2-a administrare a vaccinului anti-COVID-19 au aparut reactiile adverse atribuite acestuia?

- ○ Nu se aplica, nu am primit inca a doua doza a vaccinului
- ○ Nu se aplica, nu am avut reactii adverse
- ○ Mai putin de 12 ore
- ○ 12-24 ore
- ○ 24-48 ore
- ○ 2-4 zile
- ○ 4-7 zile
- ○ Mai mult de 7 zile
- ○ Altceva (va rugam precizati)

* 18. Ce durata au avut reactiile adverse aparute dupa cea de a 2-a adminstrare a vaccinului anti-COVID-19 ?

- ◯ Nu se aplica, nu am primit inca a doua doza a vaccinului
- ◯ Nu se aplica, nu am avut reactii adverse
- ◯ Mai putin de 24 ore
- ◯ 1-3 zile
- ◯ 3-7 zile
- ◯ 1-2 saptamani
- ◯ 2-4 saptamani
- ◯ 1-2 luni
- ◯ Mai mult de 2 luni
- ◯ Altceva (va rugam precizati)

* 19. Ce medicamente ati luat ca tratament pentru reactiile adverse aparute dupa cea de a 2-a administrare a vaccinului anti-COVID-19 ?

- ☐ Nu se aplica, nu am primit inca a doua doza a vaccinului
- ☐ Nu se aplica, nu am avut reactii adverse
- ☐ Nimic, s-au ameliorat spontan
- ☐ Antihistaminice
- ☐ Paracetamol
- ☐ AINS
- ☐ Corticosteroizi
- ☐ Altceva (va rugam precizati)

* 20. In cazul in care ati avut reactii adverse secundare vaccinarii anti-COVID-19, ati raportat aceste reactii adverse pe platforma nationala special creata?

- ◯ Nu se aplica, nu am avut reactii adverse
- ◯ Da, am raportat reactiile adverse
- ◯ Nu, nu am raportat reactiile adverse

* 21. Credeti ca reactiile adverse aparute in contextul vaccinarii anti-COVID-19 pot necesita eliberarea de concediu medical?

- Nu, sunt reactii usoare care nu afecteaza activitatea zilnica
- Da, foarte rar ar putea fi necesara o zi de concediu medical post-vaccinare
- Da, destul de frecvent reactiile secundare aparute pot afecta activitatea zilnica pentru mai multe zile
- Da, concediul medical post-vaccinare ar trebui sa fie regula

* 22. Daca ati determinat cantitativ anticorpii neutralizanti anti-spike SARS-CoV-2 dupa al 2-lea rapel al vaccinarii anti-COVID-19, care a fost nivelul acestora, respectiv nivelul maxim in cazul determinarilor multiple?

Daca ati determinat de mai multe ori titrul anticorpilor neutralizanti, va rugam sa completati datele pentru determinarea cu titrul maxim observat.

- Nu se aplica, nu am determinat niciodata anticorpii neutralizanti anti-spike SARS-CoV2
- Am determinat, titrul a fost negativ, sub limita superioara a normalului
- Titru pozitiv, cu nivel seric intre 1 si 100 ori limita superioara a normalului
- Titru pozitiv, de 100-1000 ori mai mare comparativ cu limita superioara a normalului
- Titru pozitiv, de 1000-5000 ori mai mare comparativ cu limita superioara a normalului
- Titru pozitiv, de 5000-10000 ori mai mare comparativ cu limita superioara a normalului
- Titru pozitiv, de mai mult de 10000 ori mai mare comparativ cu limita superioara a normalului

* 23. Daca ati determinat cantitativ anticorpii neutralizanti anti-spike SARS-CoV-2, la ce interval de timp fata de cel de al 2-lea rapel al vaccinarii anti-COVID-19 a fost determinat titrul declarat mai sus?

- Nu se aplica, nu am determinat niciodata anticorpii neutralizanti anti-spike SARS-CoV-2
- La mai putin de 2 saptamani de la al 2-lea rapel
- La 2 - 4 saptamani de la rapel
- La 1 - 2 luni de la rapel
- La 2 - 3 luni de la rapel

* 24. Ati fost diagnosticat cu infectie SARS-CoV-2 ?

- Nu, niciodata
- Da, o singura data
- Da, am avut de 2 ori COVID-19
- Altceva (va rugam precizati)

Experiența vaccinării COVID-19 pentru medicii din România

* 25. In ce moment ati fost diagnosticat/a cu COVID-19?

- ◯ Inainte de prima doza a vaccinarii anti-COVID-19
- ◯ Intre cele doua doze de vaccinare anti-COVID-19
- ◯ In primele 2 saptamani dupa a doua doza a vaccinarii anti-COVID-19
- ◯ La mai mult de 2 saptamani dupa a doua doza a vaccinarii anti-COVID-19
- ◯ Altceva (va rugam precizati)

* 26. Va rugam sa precizati in ce moment ati fost diagnosticat/a cu COVID-19?

- ◯ Martie 2020
- ◯ Aprilie 2020
- ◯ Mai 2020
- ◯ Iunie 2020
- ◯ Iulie 2020
- ◯ August 2020
- ◯ Septembrie 2020
- ◯ Octombrie 2020
- ◯ Noiembrie 2020
- ◯ Decembrie 2020
- ◯ Ianuarie 2021
- ◯ Februarie 2021
- ◯ Martie 2021
- ◯ Aprilie 2021

* 27. Daca ati avut infectie SARS-CoV2, ce forma de boala ati avut ?

- ◯ Asimptomatica
- ◯ COVID-19 forma usoara
- ◯ COVID-19 forma moderata
- ◯ COVID-19 forma severa

* 28. Daca ati avut infectie SARS-CoV-2, ce manifestari ati prezentat? (raspuns multiplu posibil)

- [ ] Astenie marcata
- [ ] Febra
- [ ] Frison
- [ ] Tuse
- [ ] Dispnee
- [ ] Anosmie/ageuzie
- [ ] Durere abdominala
- [ ] Diaree
- [ ] Anorexie
- [ ] Greata/varsaturi
- [ ] Durere toracica
- [ ] Eruptii cutanate
- [ ] Cefalee
- [ ] Vertij
- [ ] Mialgii
- [ ] Altceva (va rugam precizati)

* 29. Daca ati avut infectie SARS-CoV-2, unde ati fost tratat?

- ○ Numai la domiciliu
- ○ La spital, dar numai bilant general, fara internare
- ○ La spital, internare continua, nu am necesitat oxigenoterapie
- ○ La spital, internare continua, am necesitat oxigenoterapie, dar nu ventilatie
- ○ La spital, internare continua, inclusiv ventilatie non-invaziva
- ○ La spital, internare continua, inclusiv intubatie
- ○ Altceva (va rugam precizati)

* 30. Daca ati avut infectie SARS-CoV2, va rugam sa precizati cu care dintre urmatoarele medicamente ati fost tratat?
(raspuns multiplu posibil)

- ☐ Paracetamol
- ☐ Vitamina C
- ☐ Vitamina D
- ☐ Zinc
- ☐ Famotidina
- ☐ Quercetina
- ☐ Ivermectina
- ☐ Dexametazona
- ☐ Alt corticosteroid decat dexametazona
- ☐ Remdesivir / Veklury
- ☐ Lopinavir-Ritonavir / Kaletra
- ☐ Umifenovir / Arbidol
- ☐ Hidroxiclorochina / Plaquenil
- ☐ Azitromicina
- ☐ Tocilizumab/ RoActemra
- ☐ Anakinra/ Kineret
- ☐ Plasma de la pacienti vindecati
- ☐ Anticoagulare cu heparina cu greutate moleculara mica
- ☐ Anticoagulate cu NOAC
- ☐ Antiagregant
- ☐ Altceva (va rugam precizati)
