# Supplementary material for: Physicians’ Experience with COVID-19 Vaccination: A Survey Study
Source: Healthcare (Basel). 2021 Dec 17;9(12):1746. doi: 10.3390/healthcare9121746 (PMC8702136; doi:10.3390/healthcare9121746)
Supplement: Supplementary file 1 [file healthcare-09-01746-s001.zip › Supplementary File S2.pdf]

Q1 Sunteti medic?

Answered: 509 Skipped: 0

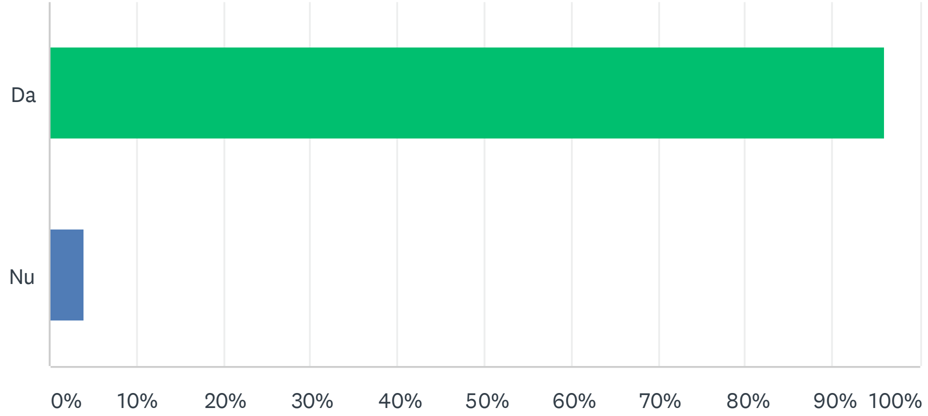

ANSWER CHOICES	RESPONSES	
Da	96.07%	489
Nu	3.93%	20
TOTAL		509

Q2 Lucrati in Romania?

Answered: 488 Skipped: 21

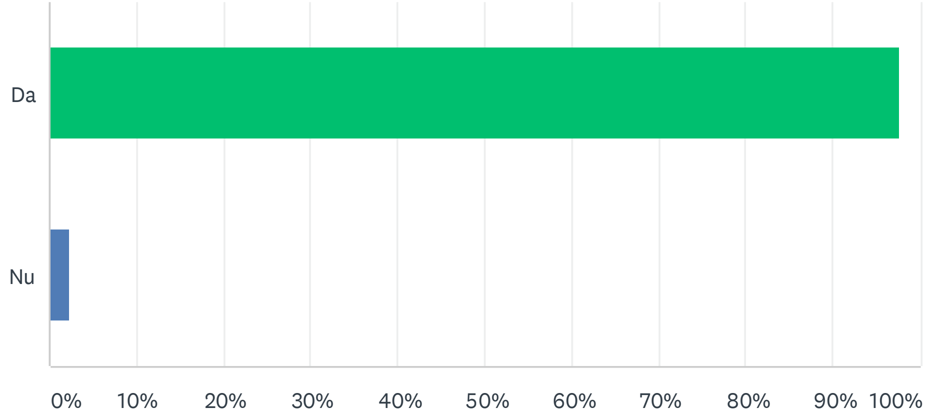

ANSWER CHOICES	RESPONSES	
Da	97.75%	477
Nu	2.25%	11
TOTAL		488

Q3 Varsta dumneavostra este:

Answered: 467 Skipped: 42

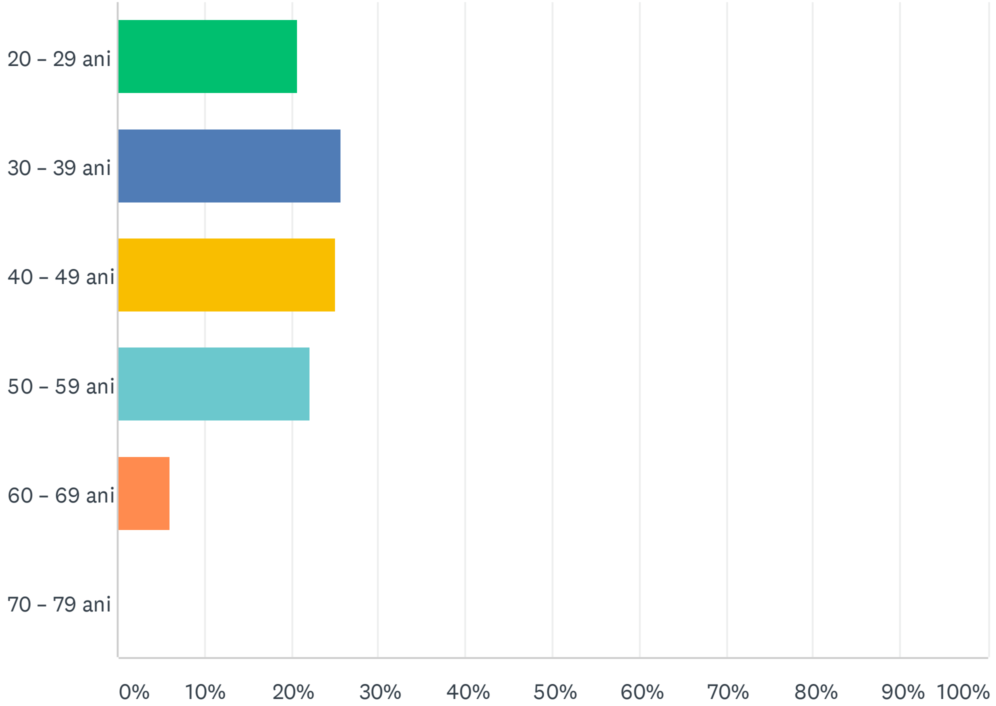

ANSWER CHOICES	RESPONSES	
20 – 29 ani	20.77%	97
30 – 39 ani	25.70%	120
40 – 49 ani	25.05%	117
50 – 59 ani	22.27%	104
60 – 69 ani	6.00%	28
70 – 79 ani	0.21%	1
TOTAL		467

Q4 Care este sexul dumneavostra?

Answered: 467 Skipped: 42

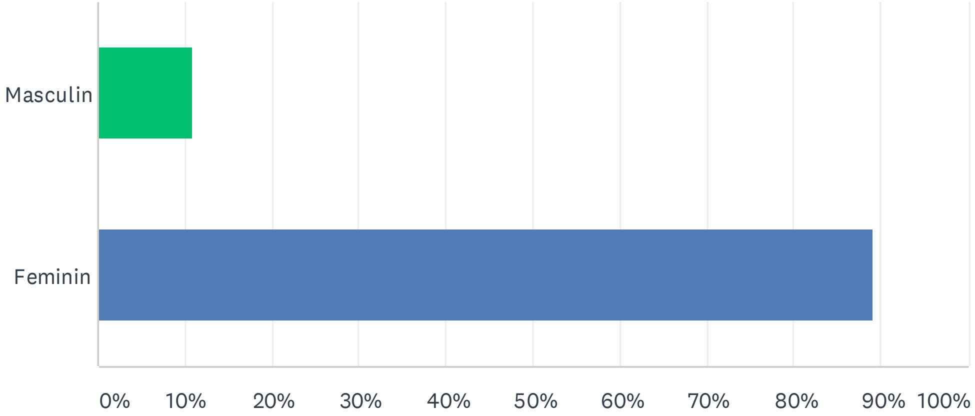

ANSWER CHOICES	RESPONSES	
Masculin	10.92%	51
Feminin	89.08%	416
TOTAL		467

Q5 Care este specialitatea in care activati?

Answered: 467 Skipped: 42

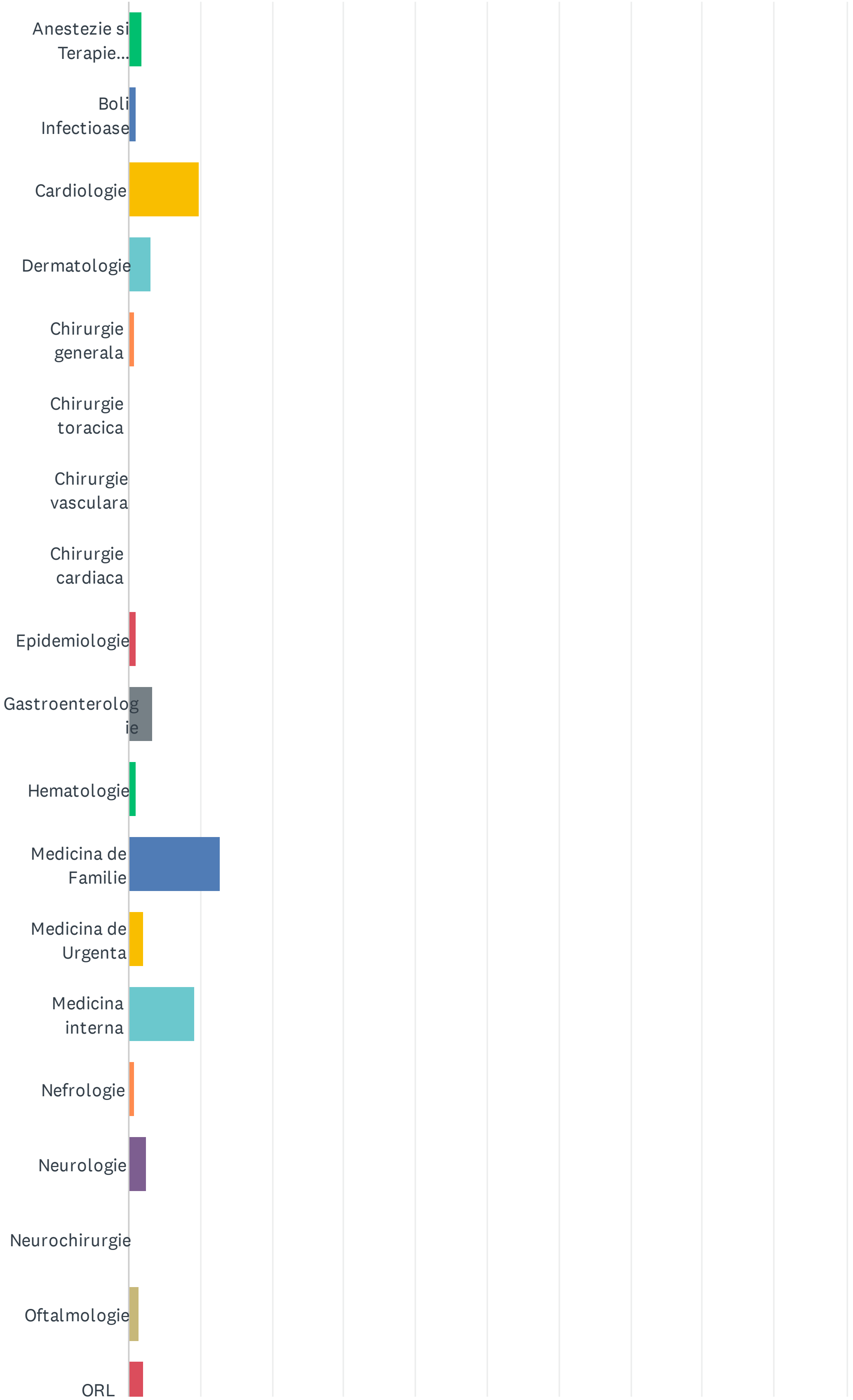

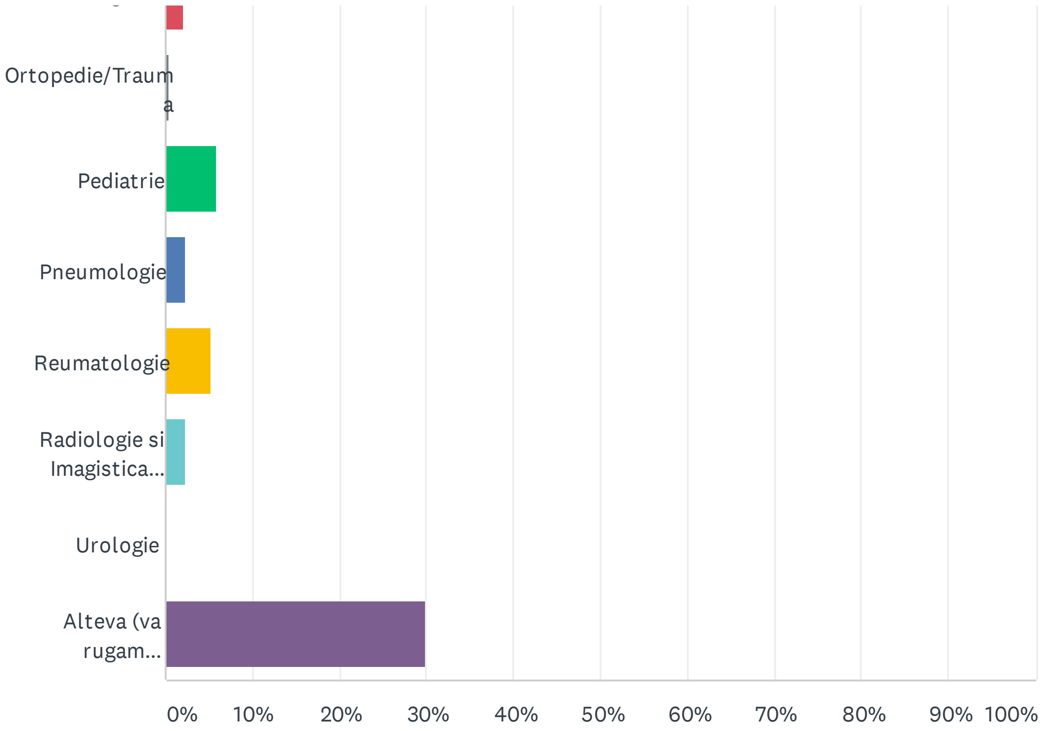
Ortopedie/Trauma
Pediatrie
Pneumologie
Reumatologie
Radiologie si Imagistica...
Urologie
Alteva (va rugam...
0%
10%
20%
30%
40%
50%
60%
70%
80%
90%
100%

ANSWER CHOICES	RESPONSES	
Anestezie si Terapie Intesiva	1.93%	9
Boli Infectioase	1.07%	5
Cardiologie	9.85%	46
Dermatologie	3.21%	15
Chirurgie generala	0.86%	4
Chirurgie toracica	0.00%	0
Chirurgie vasculara	0.21%	1
Chirurgie cardiaca	0.00%	0
Epidemiologie	1.07%	5
Gastroenterologie	3.43%	16
Hematologie	1.07%	5
Medicina de Familie	12.85%	60
Medicina de Urgenta	2.14%	10
Medicina interna	9.21%	43
Nefrologie	0.86%	4
Neurologie	2.57%	12
Neurochirurgie	0.00%	0
Oftalmologie	1.50%	7
ORL	2.14%	10
Ortopedie/Trauma	0.43%	2
Pediatrie	5.78%	27
Pneumologie	2.36%	11
Reumatologie	5.14%	24
Radiologie si Imagistica Medicala	2.36%	11
Urologie	0.00%	0
Alteva (va rugam mentionati)	29.98%	140
TOTAL		467

Q6 Care este nivelul dumneavoastra de experienta?

Answered: 466 Skipped: 43

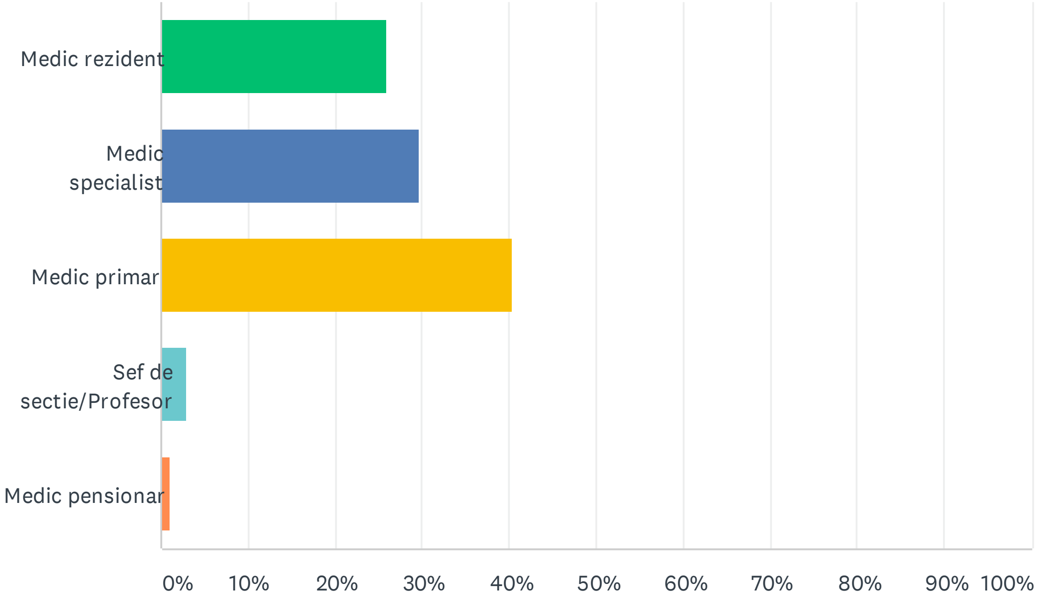

ANSWER CHOICES	RESPONSES	
Medic rezident	25.97%	121
Medic specialist	29.61%	138
Medic primar	40.34%	188
Sef de sectie/Profesor	3.00%	14
Medic pensionar	1.07%	5
TOTAL		466

Q7 Va rugam sa precizati cu care dintre urmatoarele comorbiditati ati fost diagnosticat/a:(raspuns multiplu posibil)

Answered: 466 Skipped: 43

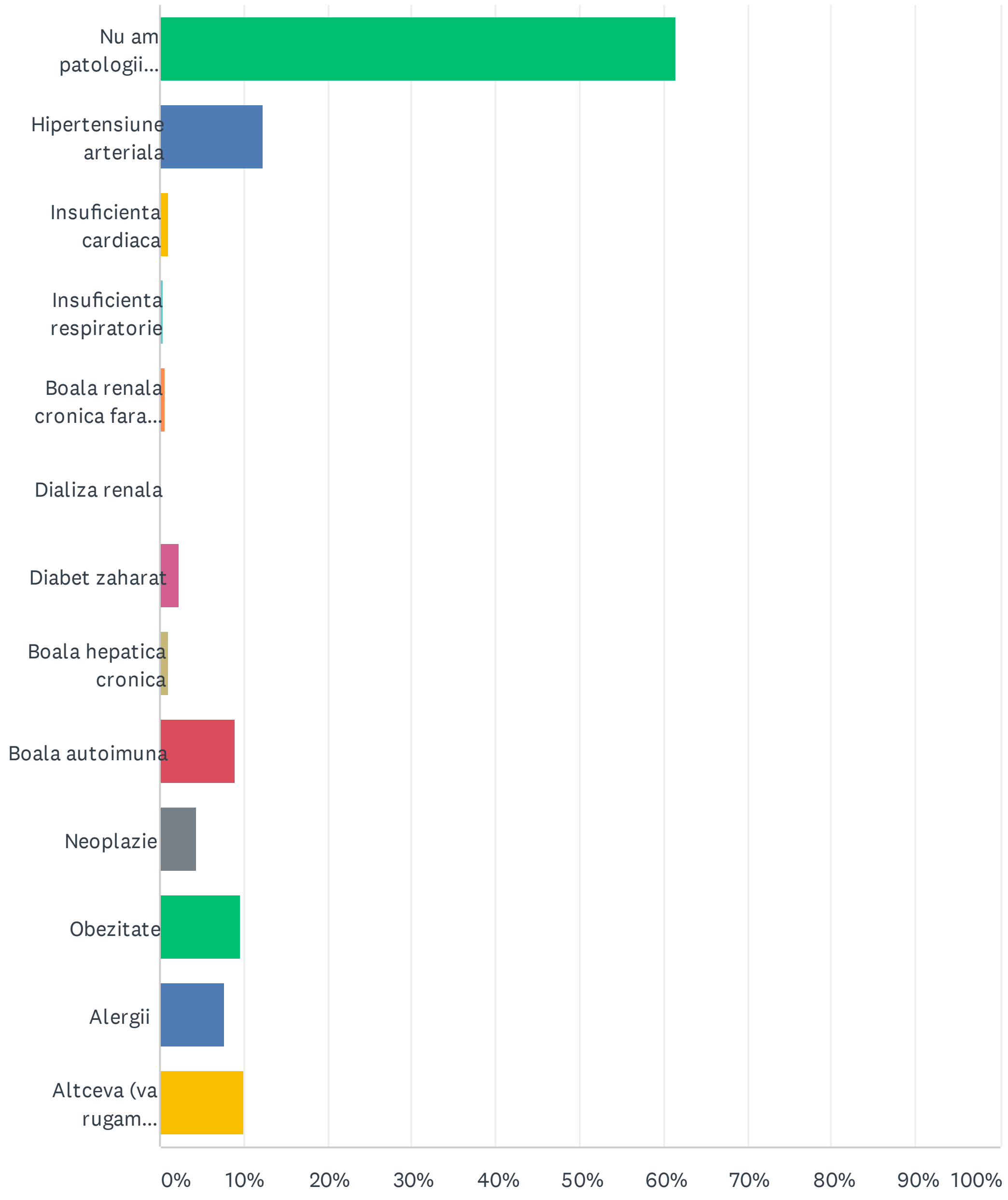

ANSWER CHOICES	RESPONSES	
Nu am patologii semnificative diagnosticate	61.59%	287
Hipertensiune arteriala	12.45%	58
Insuficienta cardiaca	1.07%	5
Insuficienta respiratorie	0.43%	2
Boala renala cronica fara dializa	0.64%	3
Dializa renala	0.00%	0
Diabet zaharat	2.36%	11
Boala hepatica cronica	1.07%	5
Boala autoimuna	9.01%	42
Neoplazie	4.29%	20
Obezitate	9.66%	45
Alergii	7.73%	36
Altceva (va rugam precizati)	10.09%	47
Total Respondents: 466		

Q8 Vi s-a administrat vaccinul anti-COVID-19?

Answered: 467 Skipped: 42

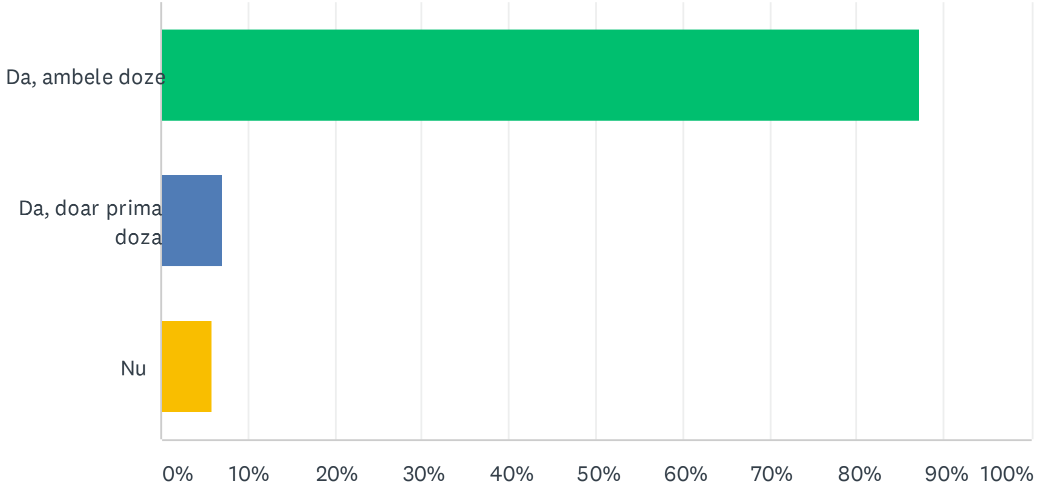

ANSWER CHOICES	RESPONSES	
Da, ambele doze	87.15%	407
Da, doar prima doza	7.07%	33
Nu	5.78%	27
TOTAL		467

Q9 Daca NU v-ati vaccinat impotriva COVID-19, care este motivul?

Answered: 24 Skipped: 485

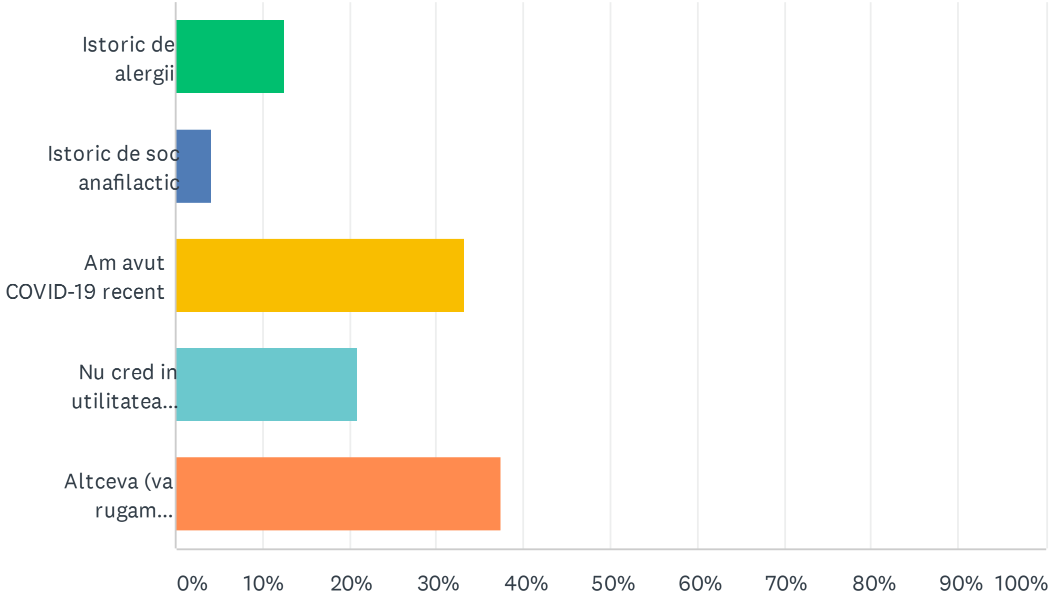

ANSWER CHOICES	RESPONSES	
Istoric de alergii	12.50%	3
Istoric de soc anafilactic	4.17%	1
Am avut COVID-19 recent	33.33%	8
Nu cred in utilitatea vaccinului	20.83%	5
Altceva (va rugam precizati)	37.50%	9
Total Respondents: 24		

Q10 Cu ce vaccin anti-COVID-19 ati fost vaccinat/a?

Answered: 420 Skipped: 89

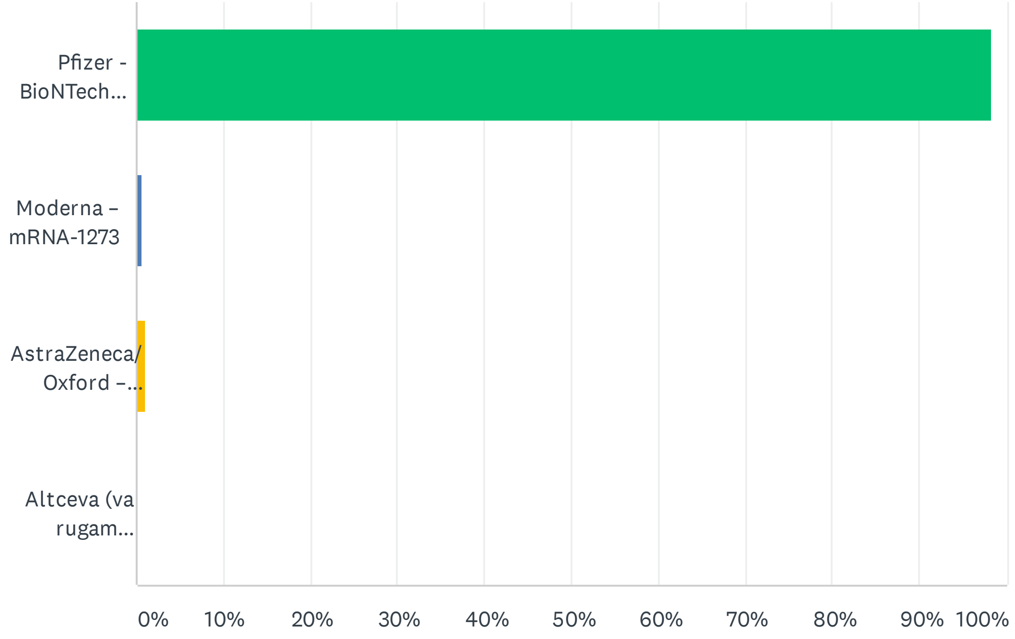

ANSWER CHOICES	RESPONSES	
Pfizer - BioNTech (Comirnaty) - BNT162b2	98.33%	413
Moderna – mRNA-1273	0.71%	3
AstraZeneca/ Oxford – AZD1222	0.95%	4
Altceva (va rugam precizati)	0.00%	0
TOTAL		420

Q11 Cand ati fost vaccinat/a impotriva COVID-19 (prima doza)?

Answered: 420 Skipped: 89

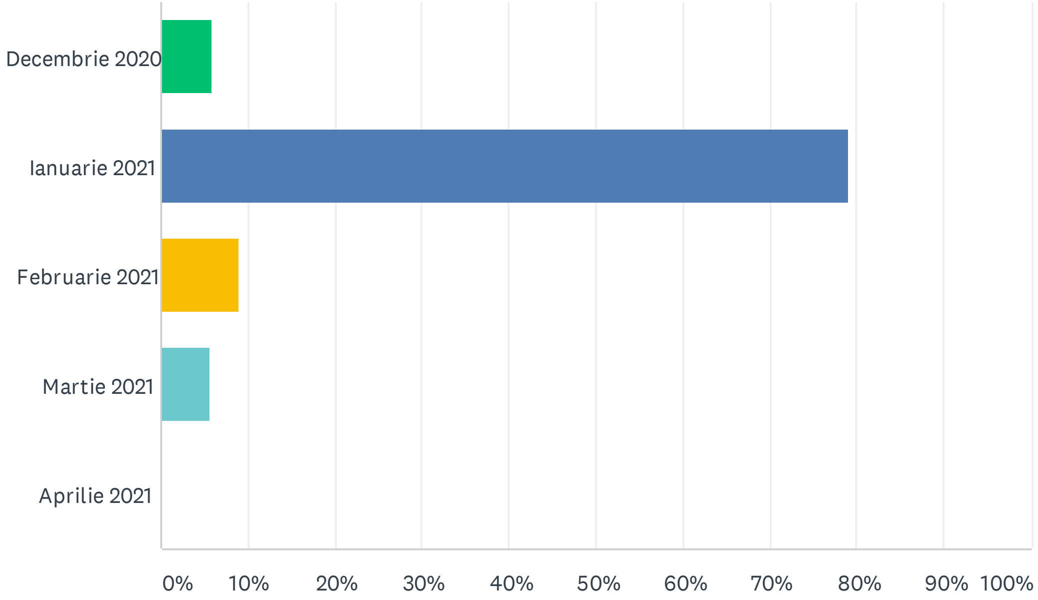

ANSWER CHOICES	RESPONSES	
Decembrie 2020	5.95%	25
Ianuarie 2021	79.05%	332
Februarie 2021	9.05%	38
Martie 2021	5.71%	24
Aprilie 2021	0.24%	1
TOTAL		420

Q12 Inainte de administrarea vaccinului anti-COVID-19 +/- inaintea aparitiei oricarei reactii adverse, ati luat vreun tratament pentru a preveni eventualele reactii adverse secundare vaccinarii? (raspuns multiplu posibil)

Answered: 420 Skipped: 89

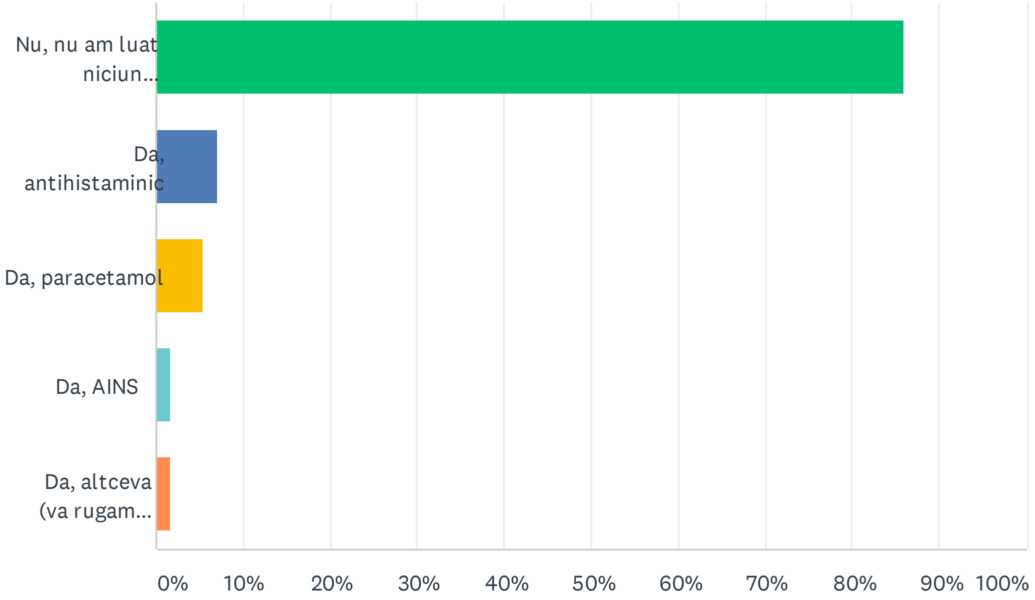

ANSWER CHOICES	RESPONSES	
Nu, nu am luat niciun medicament	85.95%	361
Da, antihistaminic	7.14%	30
Da, paracetamol	5.48%	23
Da, AINS	1.67%	7
Da, altceva (va rugam precizati)	1.67%	7
Total Respondents: 420		

Q13 Dupa administrarea primei doze a vaccinului anti-COVID-19 ce reactii adverse ati prezentat?(raspuns multiplu posibil)

Answered: 420 Skipped: 89

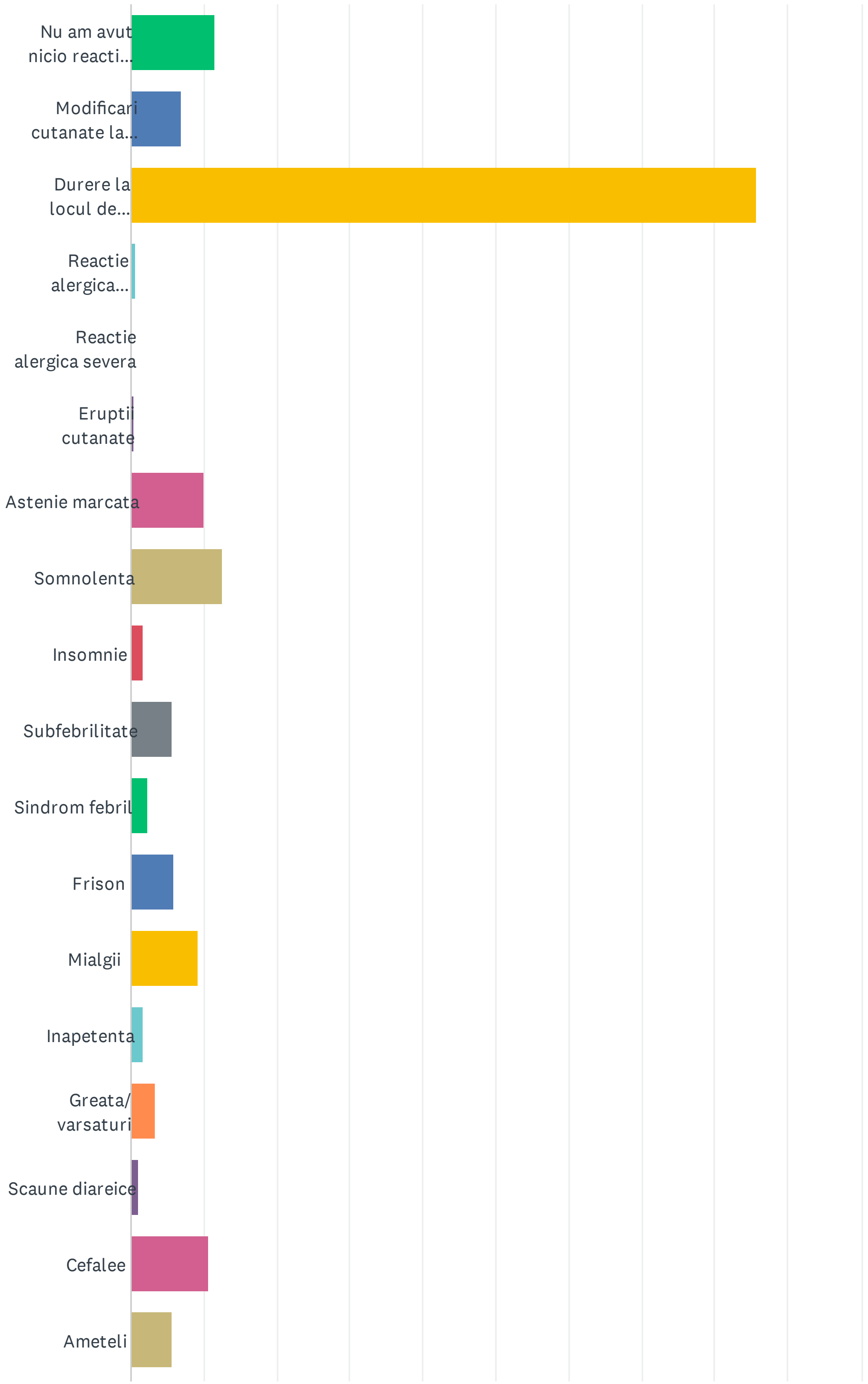

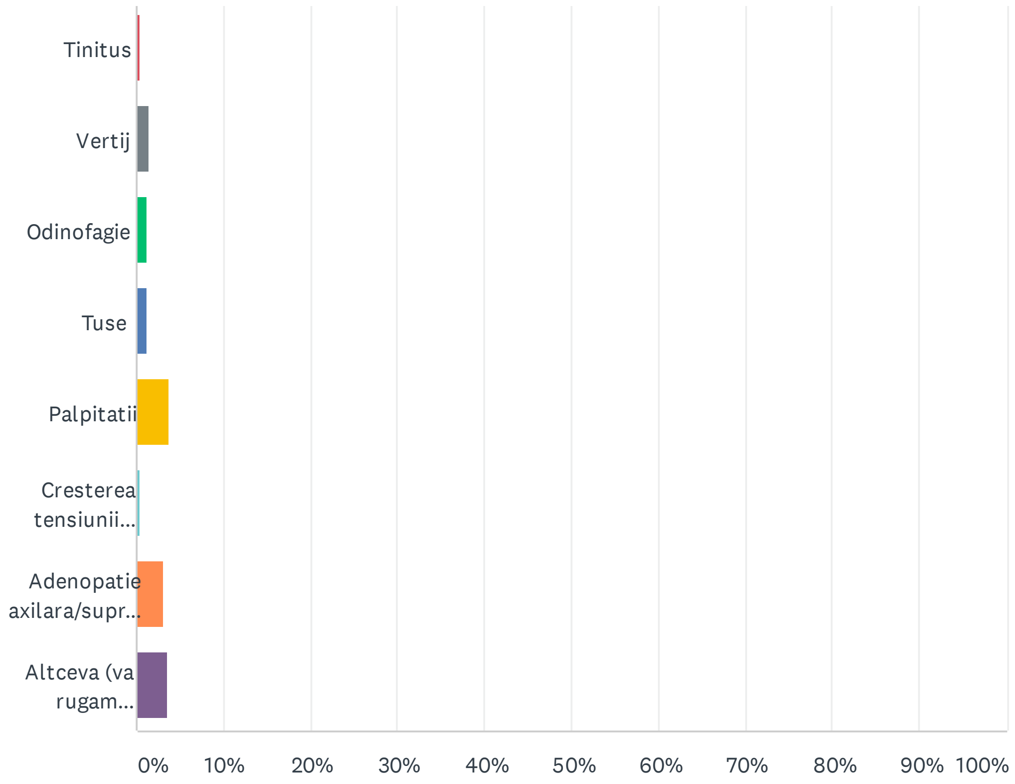
Tinitus
Vertij
Odinofagie
Tuse
Palpitatii
Cresterea tensiunii...
Adenopatie axilara/supr...
Altceva (va rugam...
0%
10%
20%
30%
40%
50%
60%
70%
80%
90%
100%

ANSWER CHOICES	RESPONSES	
Nu am avut nicio reactie adversa	11.43%	48
Modificari cutanate la locul de injectare	6.90%	29
Durere la locul de injectare	85.71%	360
Reactie alergica usoara-moderata	0.71%	3
Reactie alergica severa	0.00%	0
Eruptii cutanate	0.48%	2
Astenie marcata	10.00%	42
Somnolenta	12.62%	53
Insomnie	1.67%	7
Subfebrilitate	5.71%	24
Sindrom febril	2.38%	10
Frison	5.95%	25
Mialgii	9.29%	39
Inapetenta	1.67%	7
Greata/ varsaturi	3.33%	14
Scaune diareice	0.95%	4
Cefalee	10.71%	45
Ameteli	5.71%	24
Tinitus	0.48%	2
Vertij	1.43%	6
Odinofagie	1.19%	5
Tuse	1.19%	5
Palpitatii	3.81%	16
Cresterea tensiunii arteriale	0.48%	2
Adenopatie axilara/supraclaviculara	3.10%	13
Altceva (va rugam precizati)	3.57%	15
Total Respondents: 420		

Q14 La ce interval dupa prima adminstrare a vaccinului anti-COVID-19 au aparut reactiile adverse atribuite acestuia?

Answered: 420 Skipped: 89

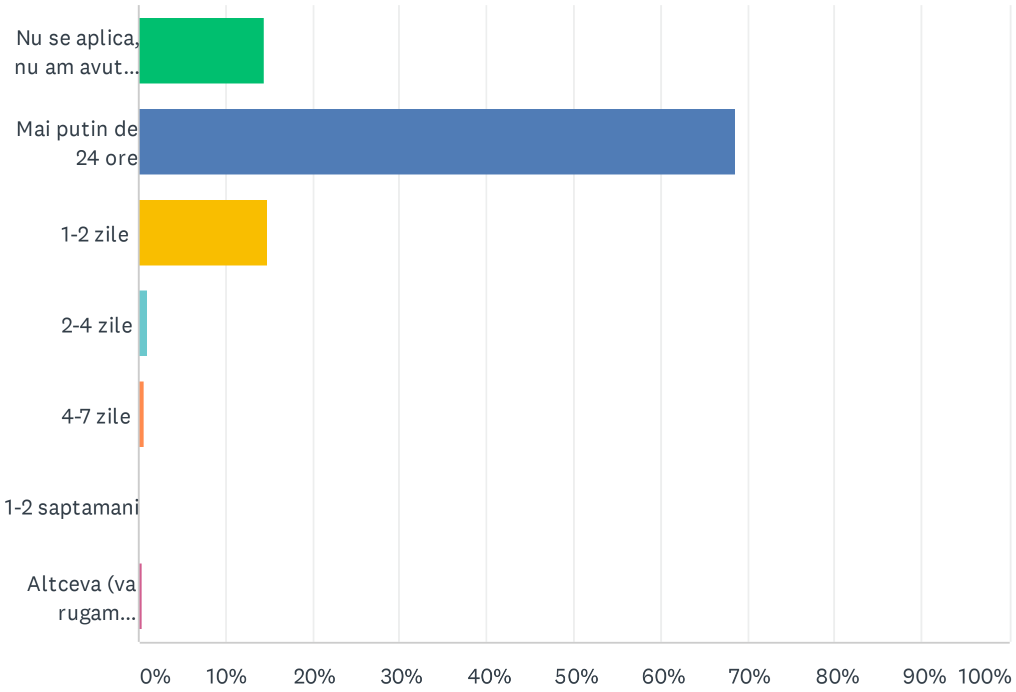

ANSWER CHOICES	RESPONSES	
Nu se aplica, nu am avut reactii adverse	14.52%	61
Mai putin de 24 ore	68.57%	288
1-2 zile	14.76%	62
2-4 zile	0.95%	4
4-7 zile	0.71%	3
1-2 saptamani	0.00%	0
Altceva (va rugam precizati)	0.48%	2
TOTAL		420

Q15 Ce medicamente ati luat ca tratament pentru a trata reactiile adverse aparute dupa prima administrare a vaccinului anti-COVID-19? (raspuns multiplu posibil)

Answered: 420 Skipped: 89

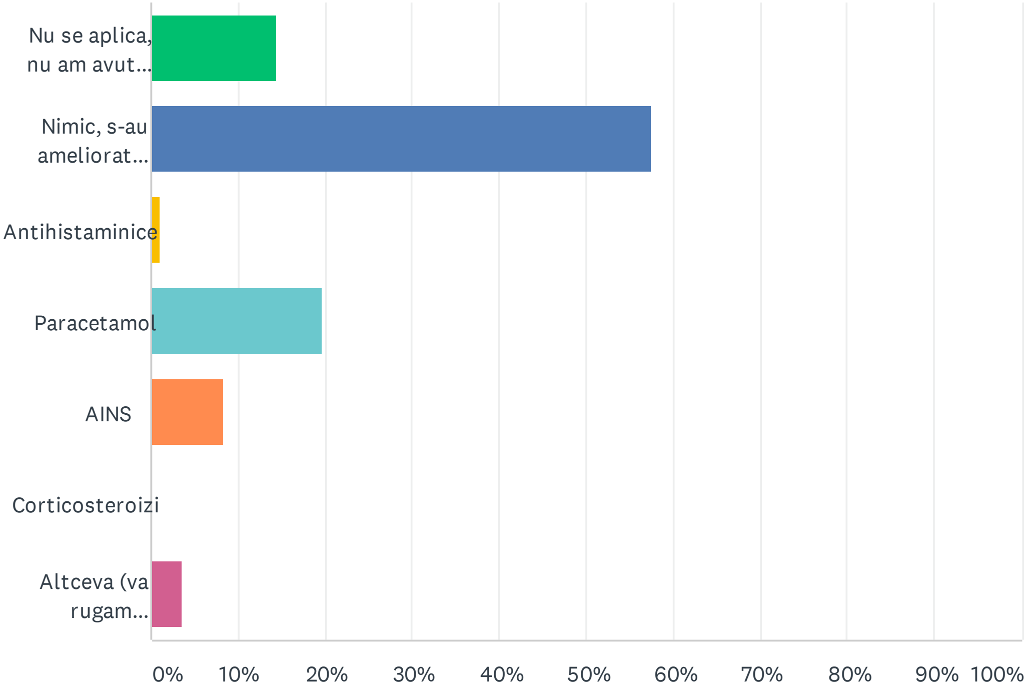

ANSWER CHOICES	RESPONSES	
Nu se aplica, nu am avut reactii adverse	14.52%	61
Nimic, s-au ameliorat spontan	57.62%	242
Antihistaminice	0.95%	4
Paracetamol	19.76%	83
AINS	8.33%	35
Corticosteroizi	0.24%	1
Altceva (va rugam precizati)	3.57%	15
Total Respondents: 420		

Q16 Dupa administrarea celei de a 2-a doze a vaccinului anti-COVID-19 ce reactii adverse ati prezentat?(raspuns multiplu posibil)

Answered: 419 Skipped: 90

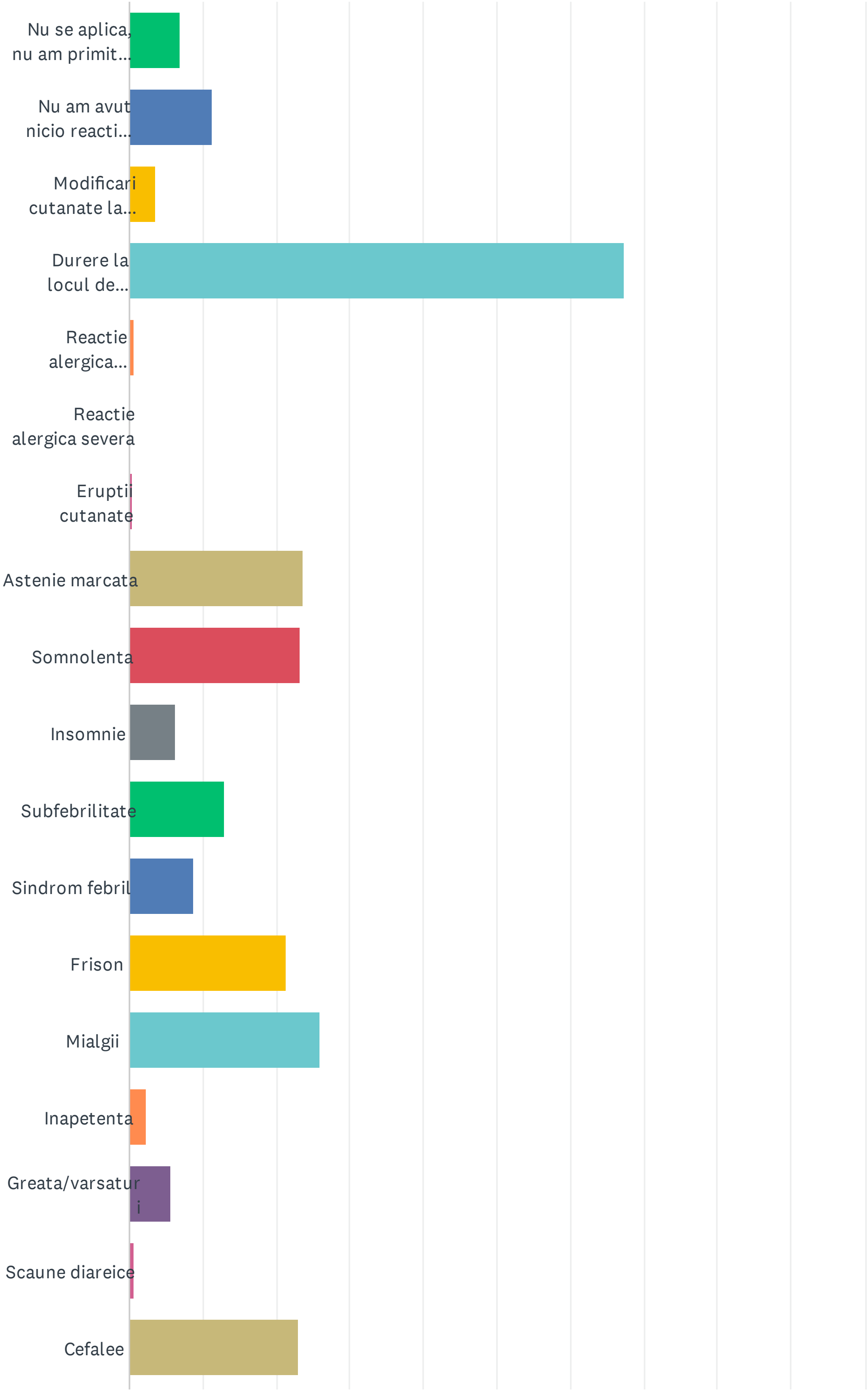

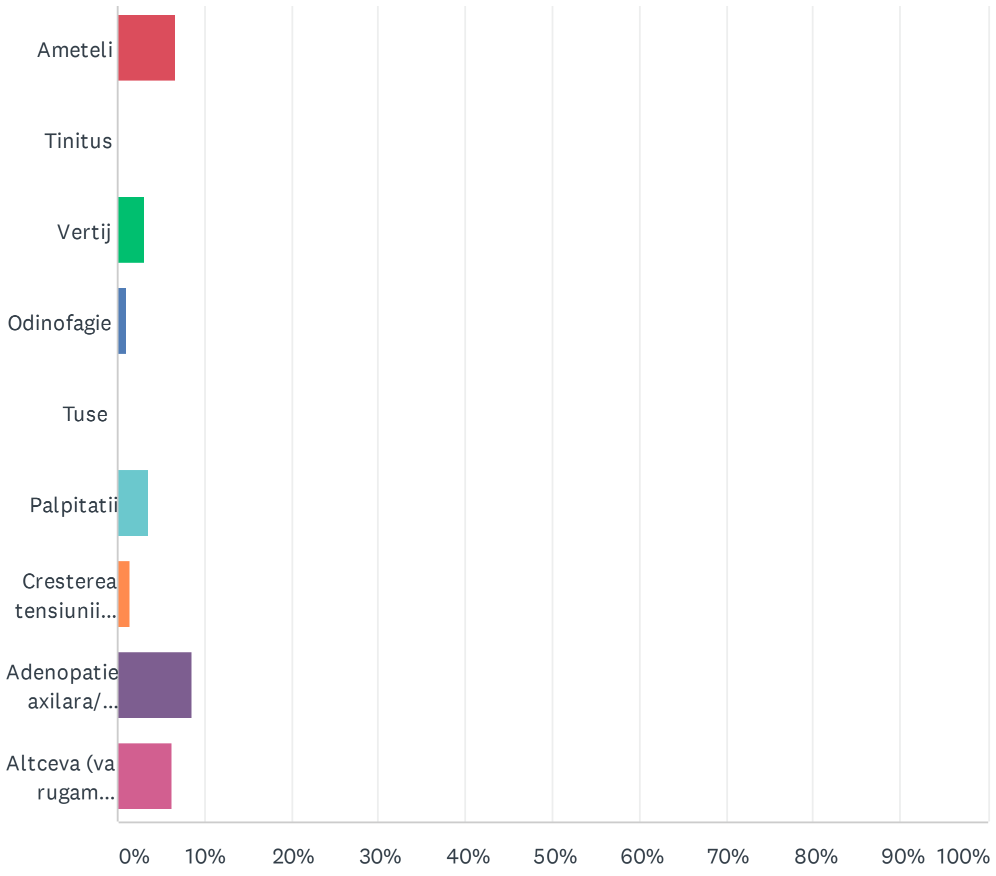
Ameteli
Tinitus
Vertij
Odinofagie
Tuse
Palpitatii
Cresterea tensiunii...
Adenopatie axilara/...
Altceva (va rugam...
0%
10%
20%
30%
40%
50%
60%
70%
80%
90%
100%

ANSWER CHOICES	RESPONSES	
Nu se aplica, nu am primit inca a doua doza a vaccinului	6.92%	29
Nu am avut nicio reactie adversa	11.22%	47
Modificari cutanate la locul de injectare	3.58%	15
Durere la locul de injectare	67.30%	282
Reactie alergica usoara-moderata	0.72%	3
Reactie alergica severa	0.00%	0
Eruptii cutanate	0.48%	2
Astenie marcata	23.63%	99
Somnolenta	23.15%	97
Insomnie	6.21%	26
Subfebrilitate	12.89%	54
Sindrom febril	8.83%	37
Frison	21.24%	89
Mialgii	26.01%	109
Inapetenta	2.39%	10
Greata/varsaturi	5.73%	24
Scaune diareice	0.72%	3
Cefalee	22.91%	96
Ameteli	6.68%	28
Tinitus	0.24%	1
Vertij	3.10%	13
Odinofagie	0.95%	4
Tuse	0.24%	1
Palpitatii	3.58%	15
Cresterea tensiunii arteriale	1.43%	6
Adenopatie axilara/ supraclaviculara	8.59%	36
Altceva (va rugam precizati)	6.21%	26
Total Respondents: 419		

Q17 La ce interval dupa cea de a 2-a administrare a vaccinului anti-COVID-19 au aparut reactiile adverse atribuite acestuia?

Answered: 418 Skipped: 91

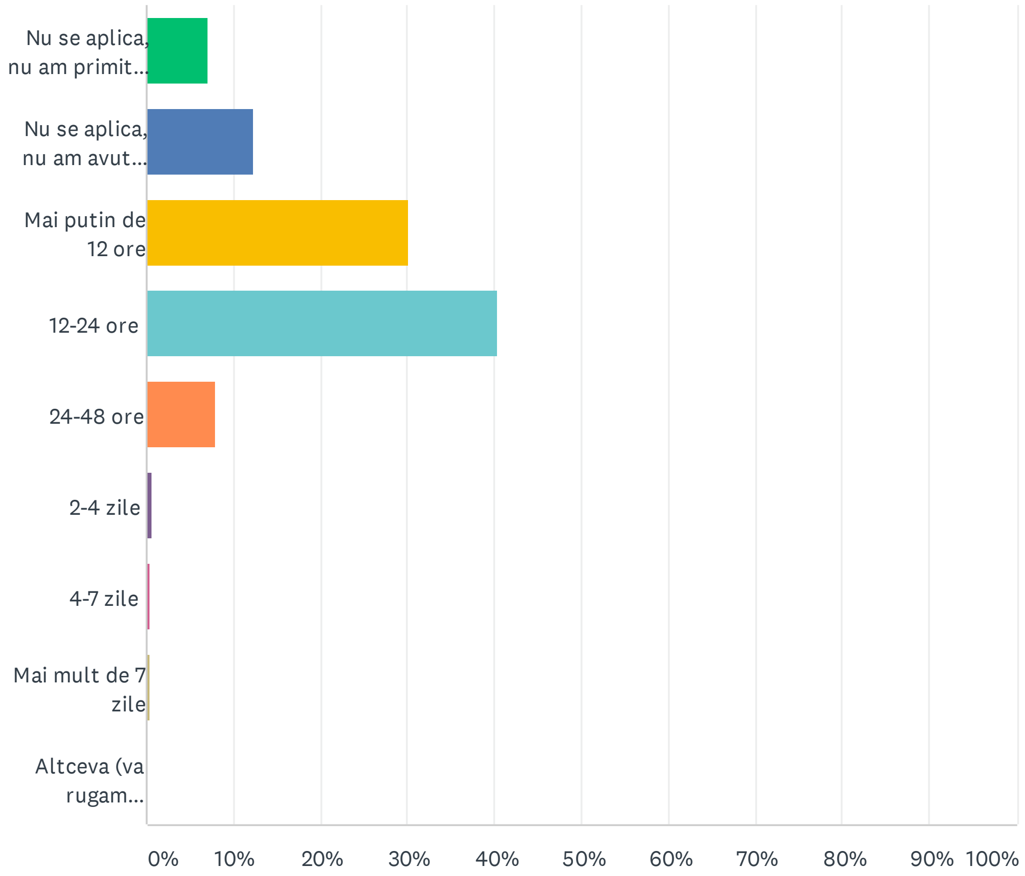

ANSWER CHOICES	RESPONSES	
Nu se aplica, nu am primit inca a doua doza a vaccinului	7.18%	30
Nu se aplica, nu am avut reactii adverse	12.44%	52
Mai putin de 12 ore	30.14%	126
12-24 ore	40.43%	169
24-48 ore	7.89%	33
2-4 zile	0.72%	3
4-7 zile	0.48%	2
Mai mult de 7 zile	0.48%	2
Altceva (va rugam precizati)	0.24%	1
TOTAL		418

Q18 Ce durata au avut reactiile adverse aparute dupa cea de a 2-a adminstrare a vaccinului anti-COVID-19 ?

Answered: 415 Skipped: 94

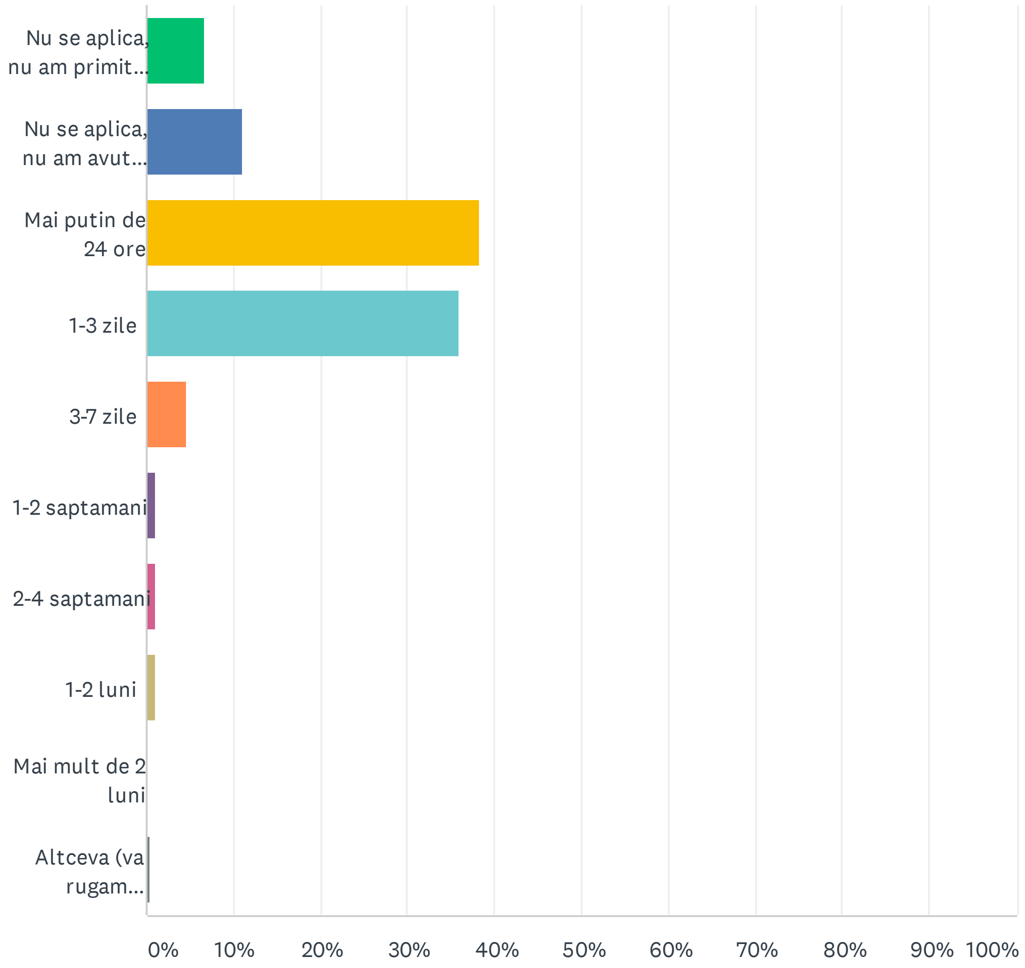

ANSWER CHOICES	RESPONSES	
Nu se aplica, nu am primit inca a doua doza a vaccinului	6.75%	28
Nu se aplica, nu am avut reactii adverse	11.08%	46
Mai putin de 24 ore	38.31%	159
1-3 zile	35.90%	149
3-7 zile	4.58%	19
1-2 saptamani	0.96%	4
2-4 saptamani	0.96%	4
1-2 luni	0.96%	4
Mai mult de 2 luni	0.00%	0
Altceva (va rugam precizati)	0.48%	2
TOTAL		415

Q19 Ce medicamente ati luat ca tratament pentru reactiile adverse aparute dupa cea de a 2-a administrare a vaccinului anti-COVID-19 ?

Answered: 417 Skipped: 92

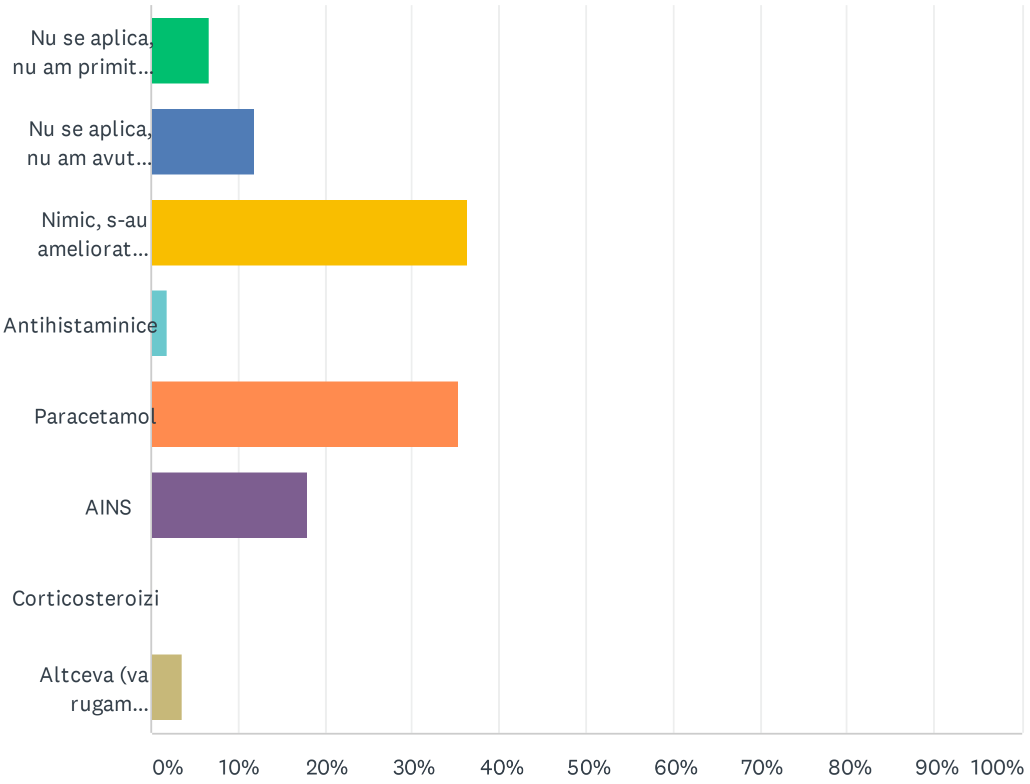

ANSWER CHOICES	RESPONSES	
Nu se aplica, nu am primit inca a doua doza a vaccinului	6.71%	28
Nu se aplica, nu am avut reactii adverse	11.99%	50
Nimic, s-au ameliorat spontan	36.45%	152
Antihistaminice	1.92%	8
Paracetamol	35.25%	147
AINS	17.99%	75
Corticosteroizi	0.24%	1
Altceva (va rugam precizati)	3.60%	15
Total Respondents: 417		

Q20 In cazul in care ati avut reactii adverse secundare vaccinarii anti-COVID-19, ati raportat aceste reactii adverse pe platforma nationala special creata?

Answered: 421 Skipped: 88

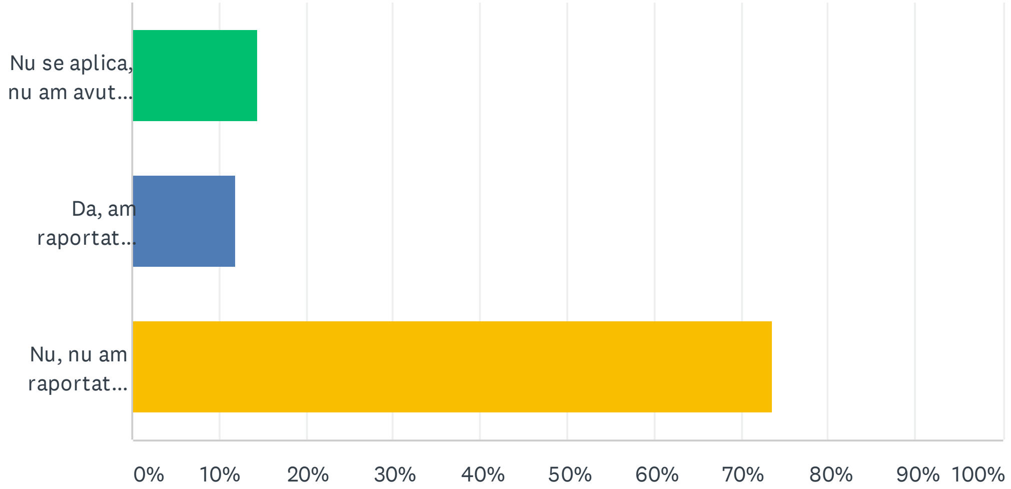

ANSWER CHOICES	RESPONSES	
Nu se aplica, nu am avut reactii adverse	14.49%	61
Da, am raportat reactiile adverse	11.88%	50
Nu, nu am raportat reactiile adverse	73.63%	310
TOTAL		421

Q21 Credeti ca reactiile adverse aparute in contextul vaccinarii anti-COVID-19 pot necesita eliberarea de concediu medical?

Answered: 421 Skipped: 88

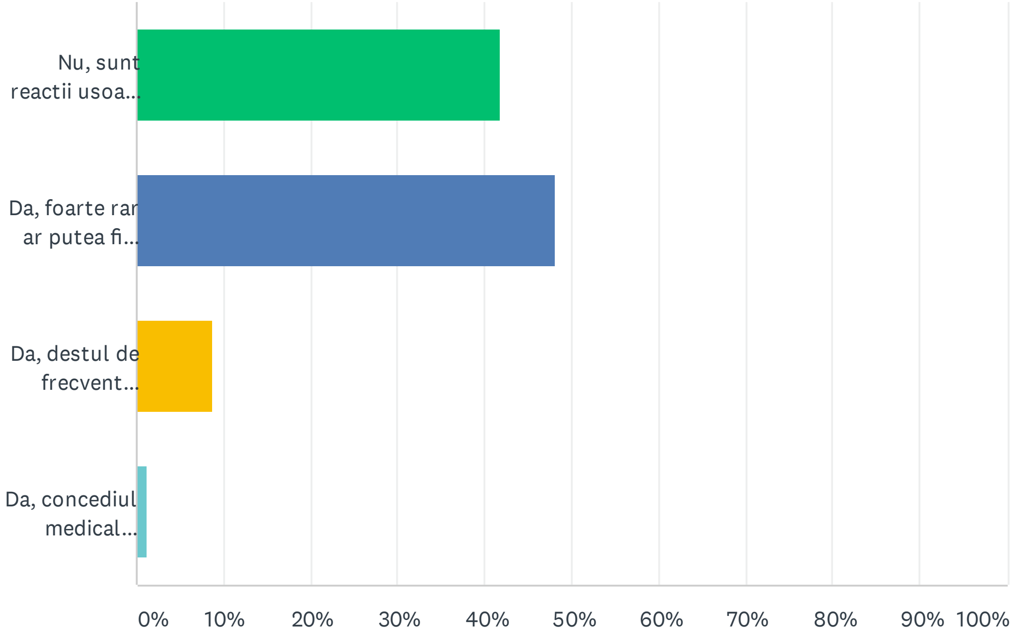

ANSWER CHOICES	RESPONSES	
Nu, sunt reactii usoare care nu afecteaza activitatea zilnica	41.81%	176
Da, foarte rar ar putea fi necesara o zi de concediu medical post-vaccinare	48.22%	203
Da, destul de frecvent reactiile secundare aparute pot afecta activitatea zilnica pentru mai multe zile	8.79%	37
Da, concediul medical post-vaccinare ar trebui sa fie regula	1.19%	5
TOTAL		421

Q22 Daca ati determinat cantitativ anticorpii neutralizanti anti-spike SARS-CoV-2 dupa al 2-lea rapel al vaccinarii anti-COVID-19, care a fost nivelul acestora, respectiv nivelul maxim in cazul determinarilor multiple? Daca ati determinat de mai multe ori titrul anticorpilor neutralizanti, va rugam sa completati datele pentru determinarea cu titrul maxim observat.

Answered: 419 Skipped: 90

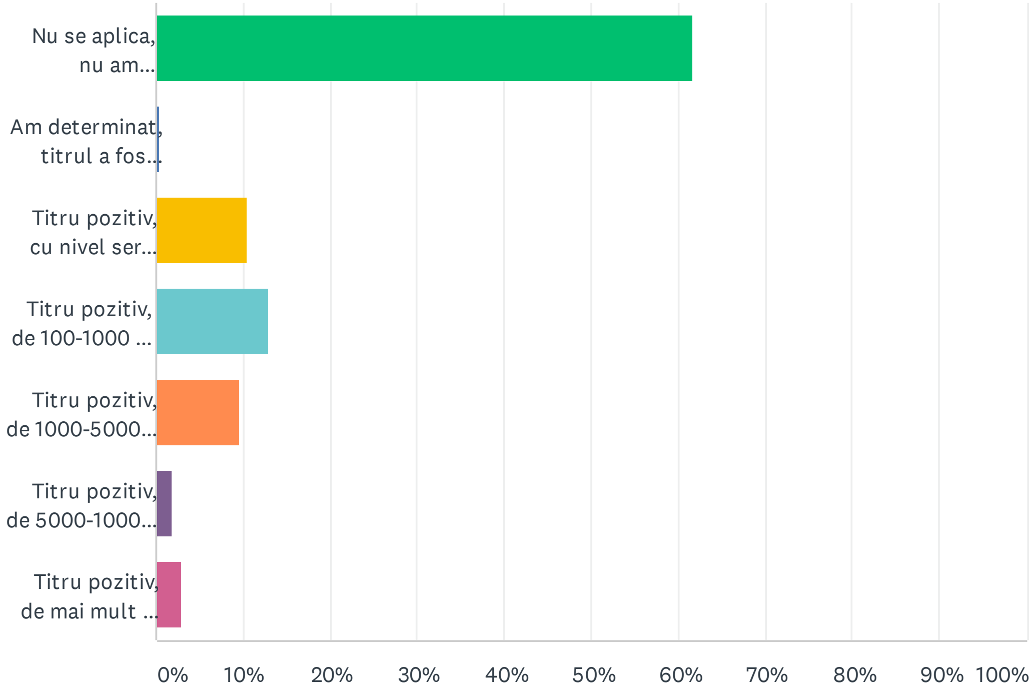

ANSWER CHOICES	RESPONSES	
Nu se aplica, nu am determinat niciodata anticorpii neutralizanti anti-spike SARS-CoV2	61.81%	259
Am determinat, titrul a fost negativ, sub limita superioara a normalului	0.48%	2
Titru pozitiv, cu nivel seric intre 1 si 100 ori limita superioara a normalului	10.50%	44
Titru pozitiv, de 100-1000 ori mai mare comparativ cu limita superioara a normalului	12.89%	54
Titru pozitiv, de 1000-5000 ori mai mare comparativ cu limita superioara a normalului	9.55%	40
Titru pozitiv, de 5000-10000 ori mai mare comparativ cu limita superioara a normalului	1.91%	8
Titru pozitiv, de mai mult de 10000 ori mai mare comparativ cu limita superioara a normalului	2.86%	12
TOTAL		419

Q23 Daca ati determinat cantitativ anticorpii neutralizanti anti-spike SARS-CoV-2, la ce interval de timp fata de cel de al 2-lea rapel al vaccinarii anti-COVID-19 a fost determinat titrul declarat mai sus?

Answered: 418 Skipped: 91

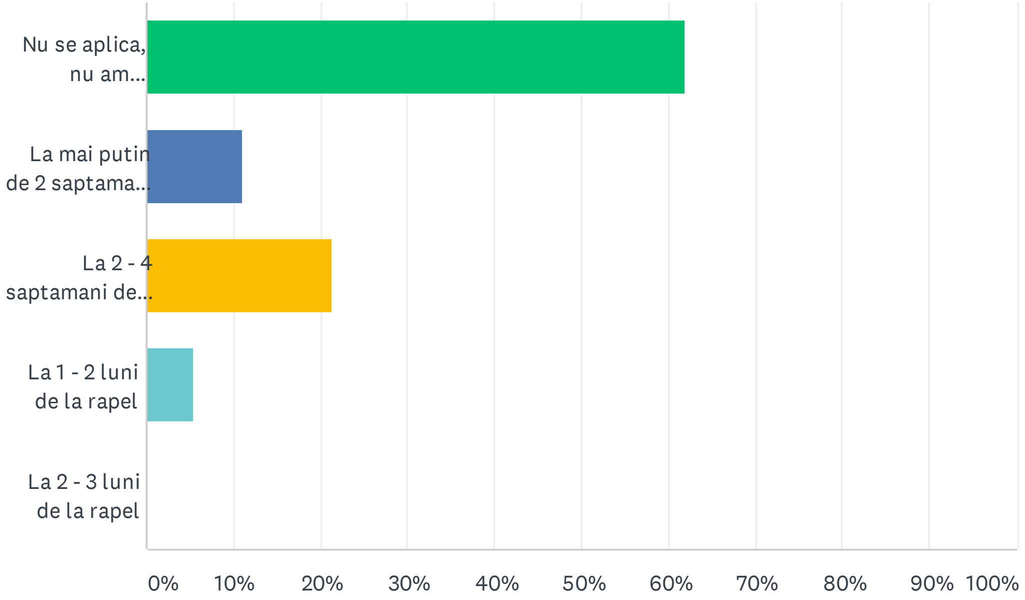

ANSWER CHOICES	RESPONSES	
Nu se aplica, nu am determinat niciodata anticorpii neutralizanti anti-spike SARS-CoV-2	61.96%	259
La mai putin de 2 saptamani de la al 2-lea rapel	11.00%	46
La 2 - 4 saptamani de la rapel	21.29%	89
La 1 - 2 luni de la rapel	5.50%	23
La 2 - 3 luni de la rapel	0.24%	1
TOTAL		418

Q24 Ati fost diagnosticat cu infectie SARS-CoV-2 ?

Answered: 421 Skipped: 88

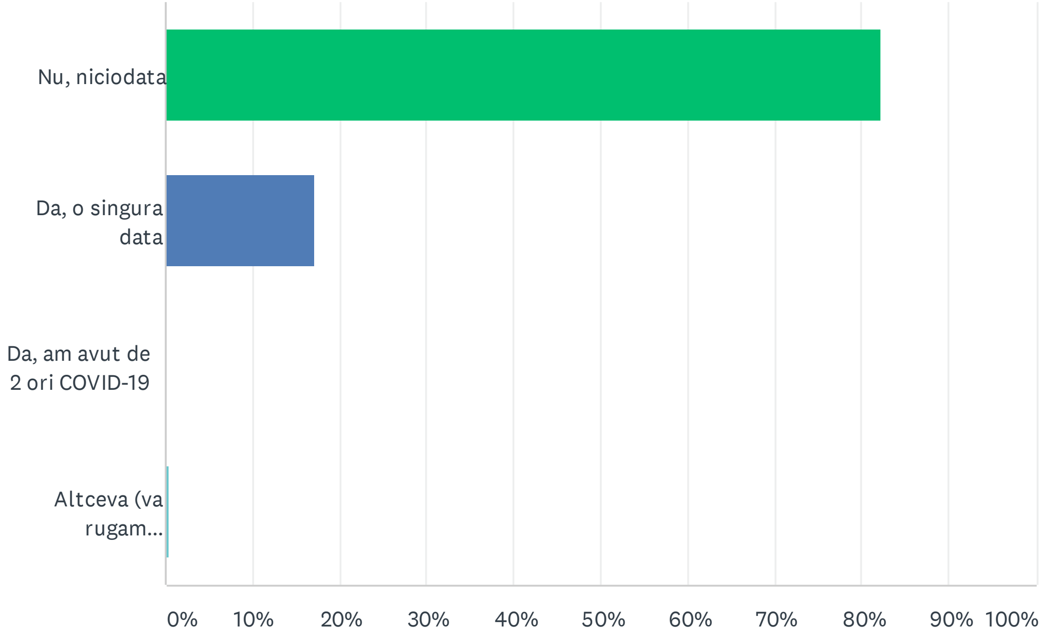

ANSWER CHOICES	RESPONSES	
Nu, niciodata	82.19%	346
Da, o singura data	17.10%	72
Da, am avut de 2 ori COVID-19	0.24%	1
Altceva (va rugam precizati)	0.48%	2
TOTAL		421

Q25 In ce moment ati fost diagnosticat/a cu COVID-19?

Answered: 72 Skipped: 437

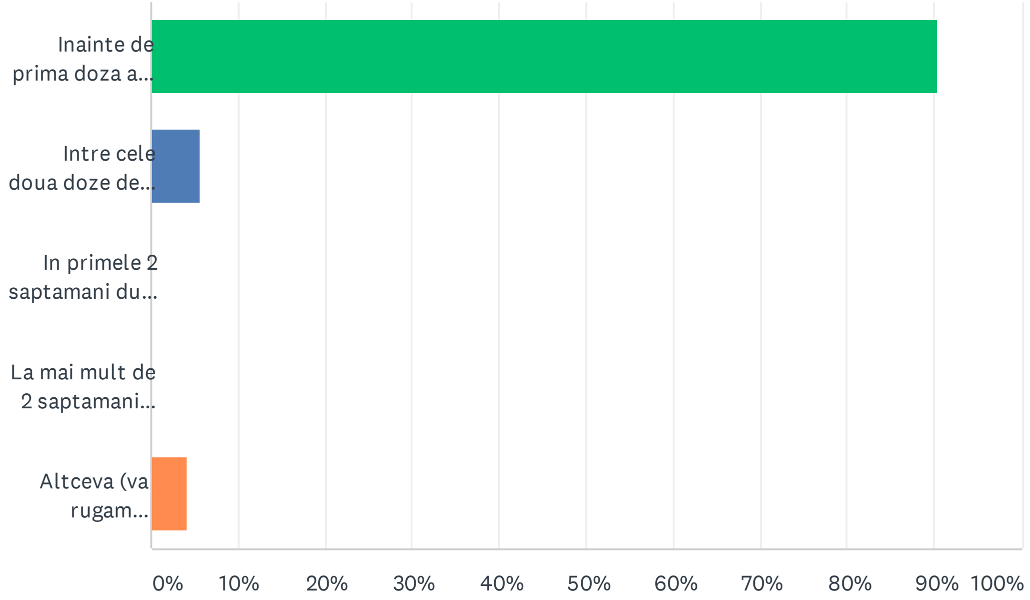

ANSWER CHOICES	RESPONSES	
Inainte de prima doza a vaccinarii anti-COVID-19	90.28%	65
Intre cele doua doze de vaccinare anti-COVID-19	5.56%	4
In primele 2 saptamani dupa a doua doza a vaccinarii anti-COVID-19	0.00%	0
La mai mult de 2 saptamani dupa a doua doza a vaccinarii anti-COVID-19	0.00%	0
Altceva (va rugam precizati)	4.17%	3
TOTAL		72

Q26 Va rugam sa precizati in ce moment ati fost diagnosticat/a cu COVID-19?

Answered: 73 Skipped: 436

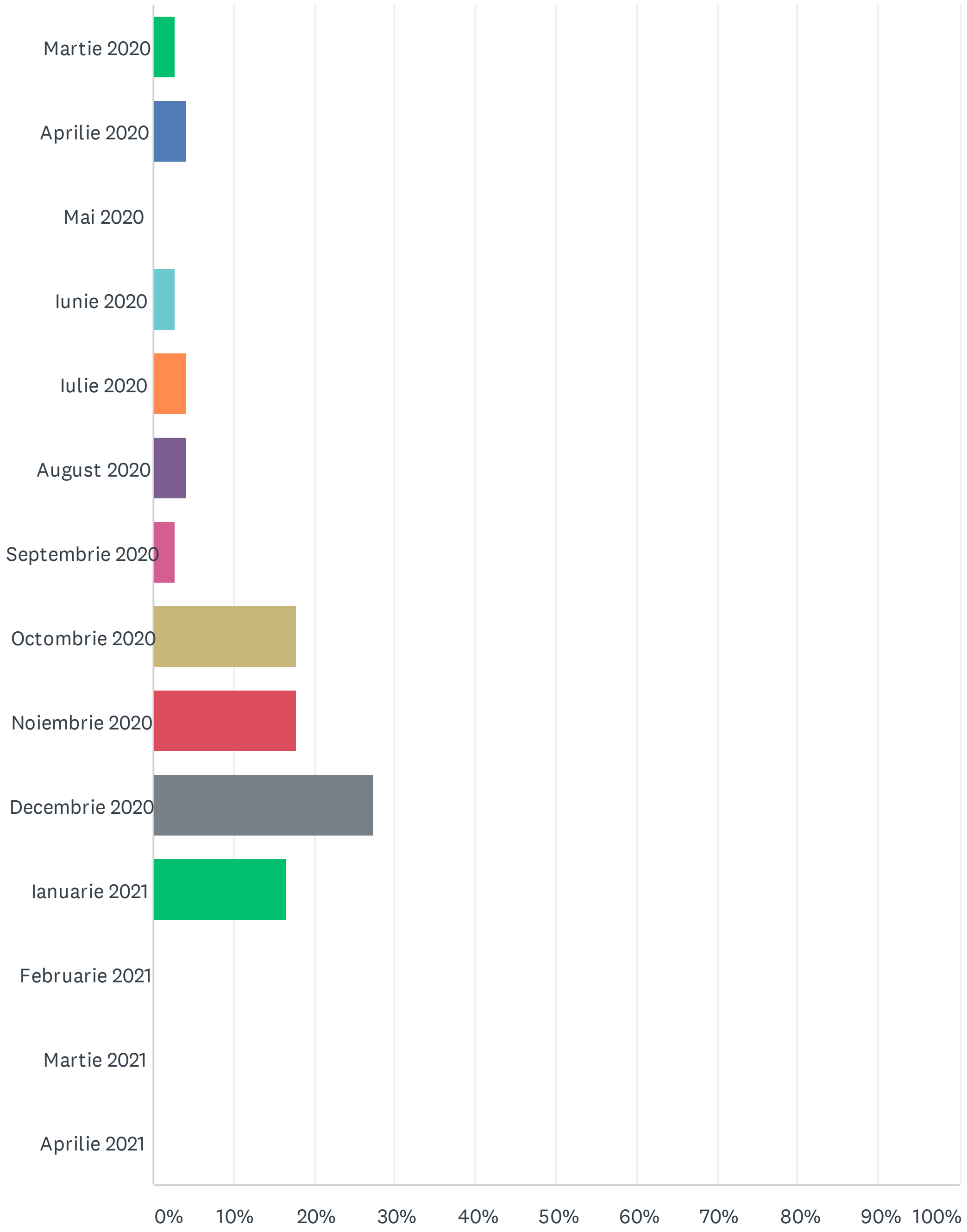

ANSWER CHOICES	RESPONSES	
Martie 2020	2.74%	2
Aprilie 2020	4.11%	3
Mai 2020	0.00%	0
Iunie 2020	2.74%	2
Iulie 2020	4.11%	3
August 2020	4.11%	3
Septembrie 2020	2.74%	2
Octombrie 2020	17.81%	13
Noiembrie 2020	17.81%	13
Decembrie 2020	27.40%	20
Ianuarie 2021	16.44%	12
Februarie 2021	0.00%	0
Martie 2021	0.00%	0
Aprilie 2021	0.00%	0
TOTAL		73

Q27 Daca ati avut infectie SARS-CoV2, ce forma de boala ati avut ?

Answered: 73 Skipped: 436

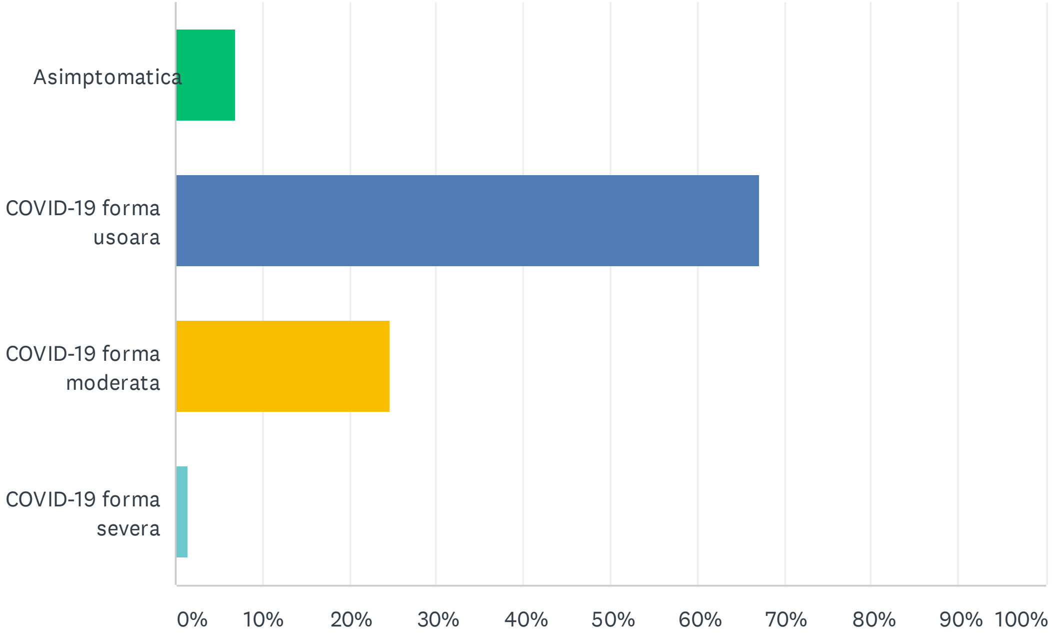

ANSWER CHOICES	RESPONSES	
Asimptomatica	6.85%	5
COVID-19 forma usoara	67.12%	49
COVID-19 forma moderata	24.66%	18
COVID-19 forma severa	1.37%	1
TOTAL		73

Q28 Daca ati avut infectie SARS-CoV-2, ce manifestari ati prezentat? (raspuns multiplu posibil)

Answered: 72 Skipped: 437

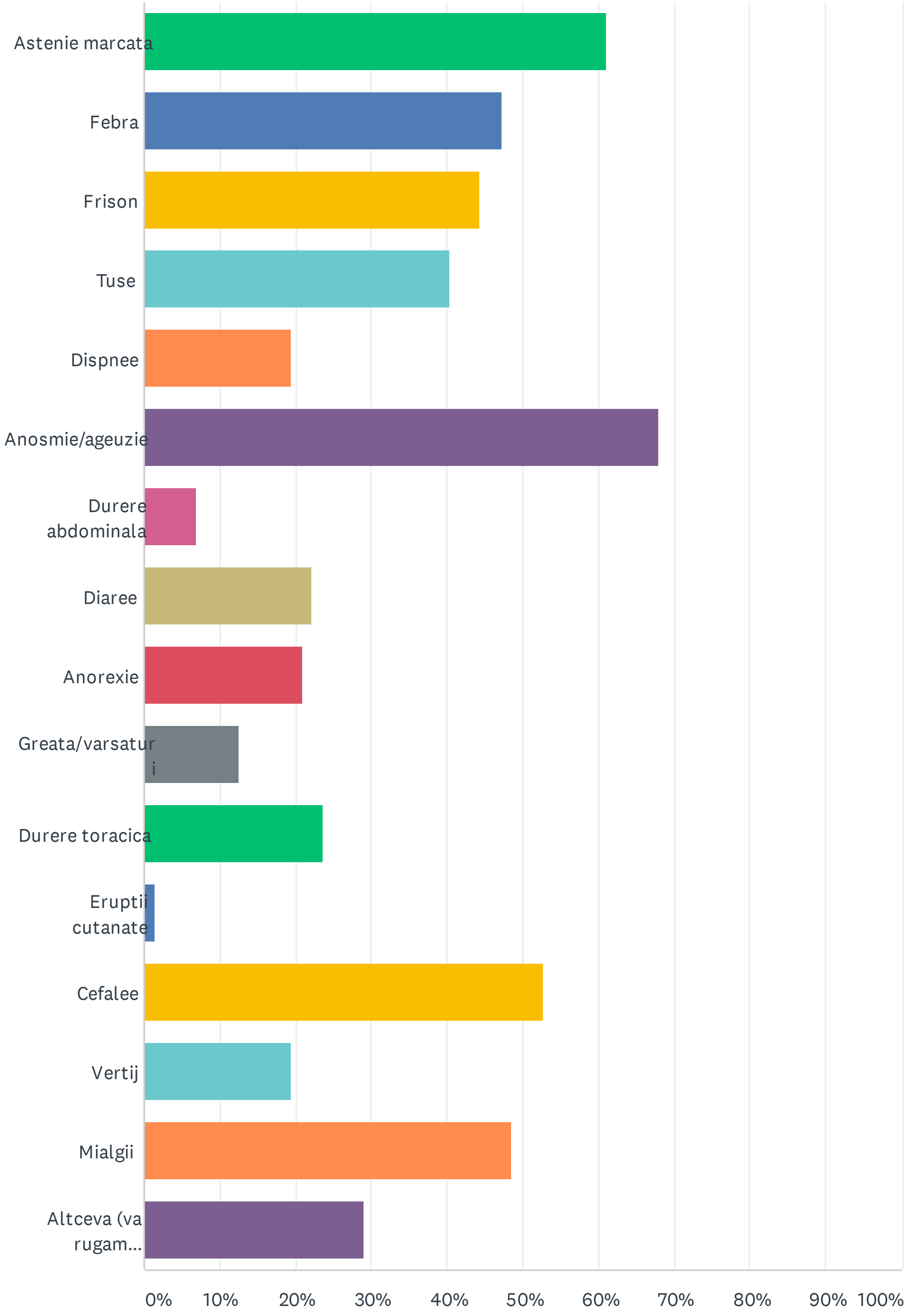

ANSWER CHOICES	RESPONSES	
Astenie marcata	61.11%	44
Febra	47.22%	34
Frison	44.44%	32
Tuse	40.28%	29
Dispnee	19.44%	14
Anosmie/ageuzie	68.06%	49
Durere abdominala	6.94%	5
Diaree	22.22%	16
Anorexie	20.83%	15
Greata/varsaturi	12.50%	9
Durere toracica	23.61%	17
Eruptii cutanate	1.39%	1
Cefalee	52.78%	38
Vertij	19.44%	14
Mialgii	48.61%	35
Altceva (va rugam precizati)	29.17%	21
Total Respondents: 72		

Q29 Daca ati avut infectie SARS-CoV-2, unde ati fost tratat?

Answered: 73 Skipped: 436

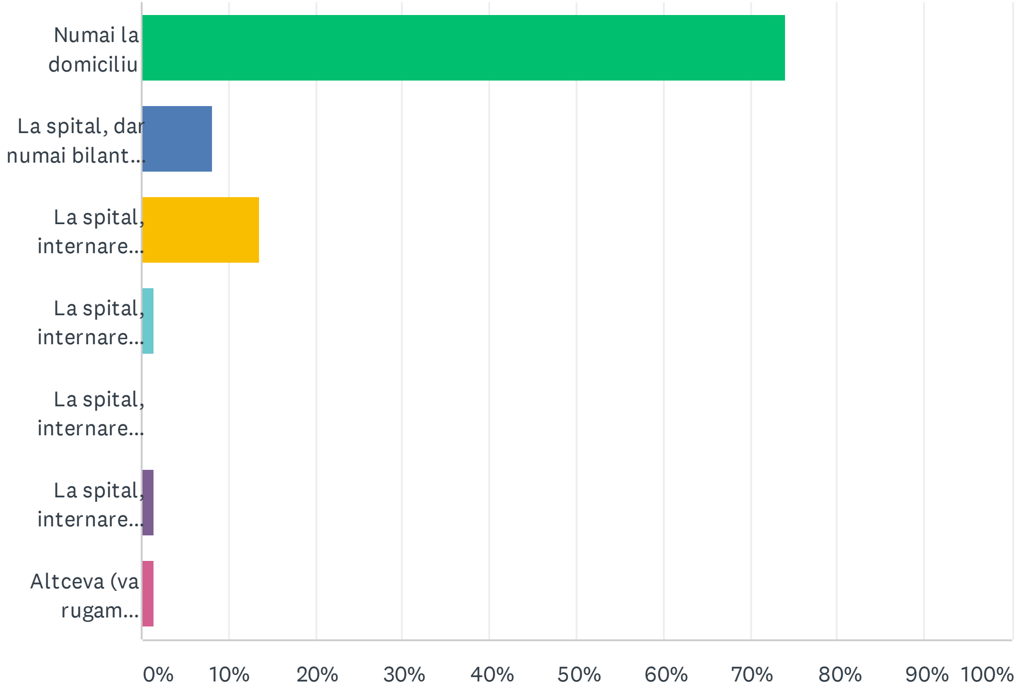

ANSWER CHOICES	RESPONSES	
Numai la domiciliu	73.97%	54
La spital, dar numai bilant general, fara internare	8.22%	6
La spital, internare continua, nu am necesitat oxigenoterapie	13.70%	10
La spital, internare continua, am necesitat oxigenoterapie, dar nu ventilatie	1.37%	1
La spital, internare continua, inclusiv ventilatie non-invaziva	0.00%	0
La spital, internare continua, inclusiv intubatie	1.37%	1
Altceva (va rugam precizati)	1.37%	1
TOTAL		73

Q30 Daca ati avut infectie SARS-CoV2, va rugam sa precizati cu care dintre urmatoarele medicamente ati fost tratat?(raspuns multiplu posibil)

Answered: 73 Skipped: 436

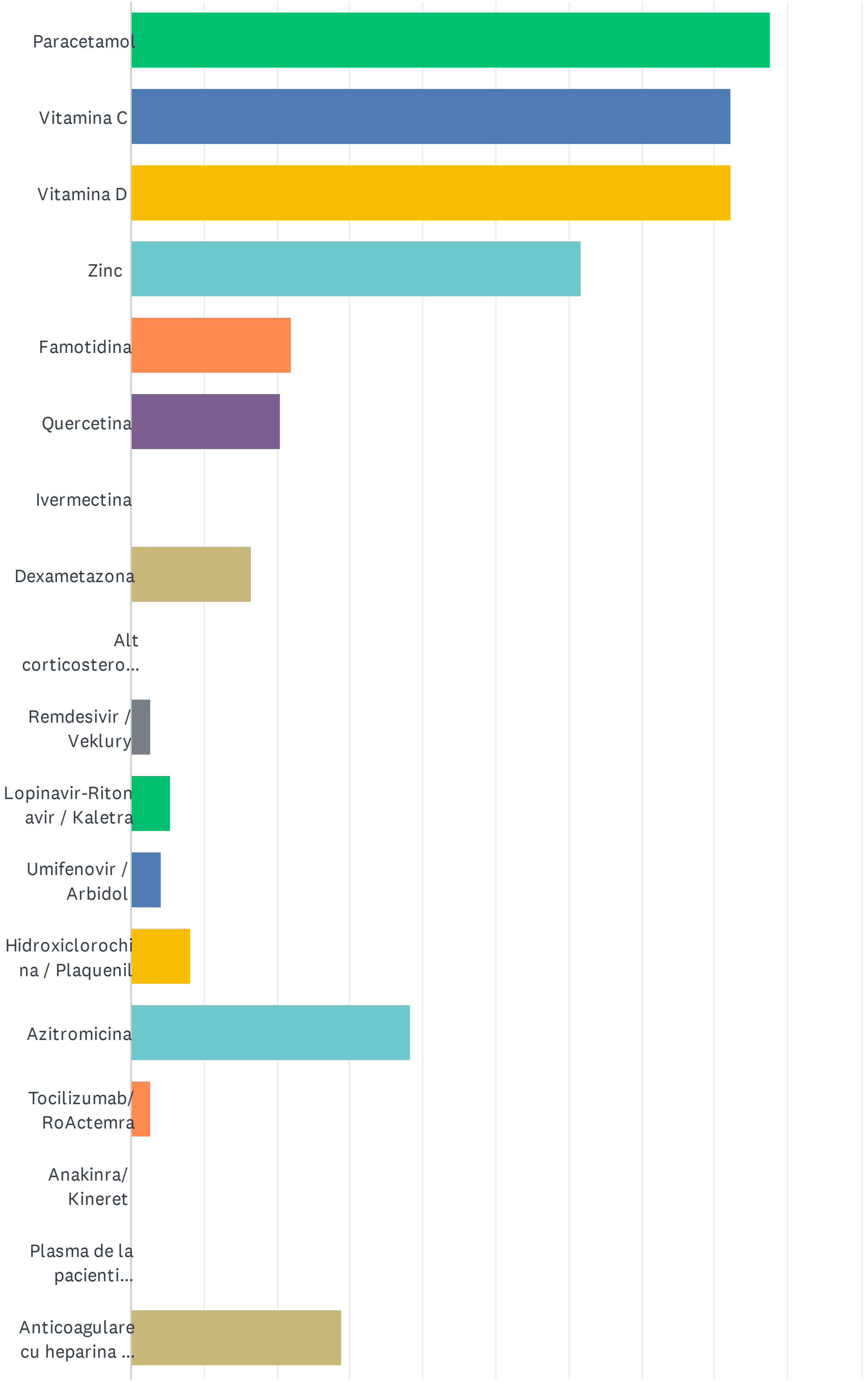

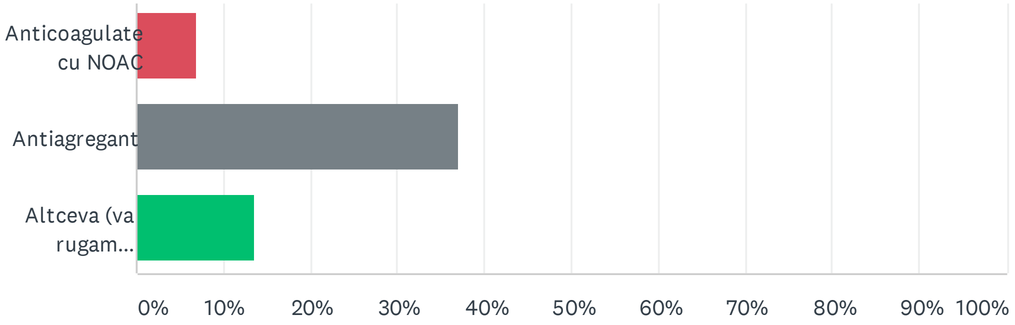

ANSWER CHOICES	RESPONSES	
Paracetamol	87.67%	64
Vitamina C	82.19%	60
Vitamina D	82.19%	60
Zinc	61.64%	45
Famotidina	21.92%	16
Quercetina	20.55%	15
Ivermectina	0.00%	0
Dexametazona	16.44%	12
Alt corticosteroid decat dexametazona	0.00%	0
Remdesivir / Veklury	2.74%	2
Lopinavir-Ritonavir / Kaletra	5.48%	4
Umifenovir / Arbidol	4.11%	3
Hidroxiclorochina / Plaquenil	8.22%	6
Azitromicina	38.36%	28
Tocilizumab/ RoActemra	2.74%	2
Anakinra/ Kineret	0.00%	0
Plasma de la pacienti vindecati	0.00%	0
Anticoagulare cu heparina cu greutate moleculara mica	28.77%	21
Anticoagulate cu NOAC	6.85%	5
Antiagregant	36.99%	27
Altceva (va rugam precizati)	13.70%	10
Total Respondents: 73		
