# Supplementary material for: Physicians’ Experience with COVID-19 Vaccination: A Survey Study
Source: Healthcare (Basel). 2021 Dec 17;9(12):1746. doi: 10.3390/healthcare9121746 (PMC8702136; doi:10.3390/healthcare9121746)
Supplement: Supplementary file 1 [file healthcare-09-01746-s001.zip › Supplementary File S3.pdf]

Tables

Supplementary Table S1. General characteristics of the physicians' respondents in the study

Characteristics	Respondents, n (%)
**Gender (Q)**	
Feminine	416/ 467 (89.08%)
Masculine	51/467 (10.92%)
**Age (years) (Q)**	
20-29	97/467 (20.77%)
30-39	120/467 (25.70%)
40-49	117/467 (25.05%)
50-59	104/467 (22.27%)
60-69	28/467 (6.00%)
70-79	1/467 (0.21%)
**Level of medical experience (Q)**	
Resident/ Intern	121/467 (25.97%)
Specialist physician < 5years experience	138/467 (29.61%)
Specialist physician > 5years experience	188/467 (40.34%)
Professor/ Chief of Department	14/467 (3.00%)
Retired physician	1/467 (1.07%)
**Medical specialty (Q)**	
Allergology	5/ 467 (1.07%)
Cardiology	46/ 467 (9.85%)
Dentistry	18/ 467 (3.85%)
Dermatology	16/ 467 (3.42%)
Diabetes	13/ 467 (2.78%)
Emergency medicine	10/ 467 (2.14%)
Endocrinology	15/ 467 (3.12%)
ENT	10/ 467 (2.14%)
Epidemiology	5/ 467 (1.07%)
Family medicine	60/ 467 (12.85%)
Gastroenterology	16/ 467 (3.43%)
General surgery	4/ 467 (0.86%)
Genetics	2/ 467 (0.42%)
Geriatrics	3/467 (0.64%)
Hematology	5/ 467 (1.07%)
Infectious diseases	5/ 467 (1.07%)
Intensive care	9/ 467 (1.93%)
Internal medicine	43/ 467 (9.21%)
Laboratory medicines	8/ 467 (1.71%)
Neonatology	2/ 467 (0.42%)
Nephrology	4/ 467 (0.86%)
Neurology	12/467 (2.57%)
Gynecology	11/ 467 (2.35%)
Oncology	4/ 467 (0.85%)

Ophthalmology	7/ 467 (1.5%)
Orthopedics	2/ 467 (0.43%)
OMF surger	2/ 467 (0.43%)
Palliatives	2/467 (0.43%)
Pathology	4/467 (0.85%)
Pediatrics	27/ 467 (5.78%)
Physical medicine	5/ 467 (1.07%)
Pneumology	11/ 467 (2.36%)
Psychiatry	21/ 467 (4.49%)
Radiology	11/ 467 (2.35%)
Rheumatology	24/ 467 (5.14%)
Vascular surgery	1/ 467 (0.21%)
**Comorbidities**	
Without significant comorbidities	287/ 466 (61.59%)
Arterial Hypertension	58/ 466 (12.45%)
Cardiac failure	5/ 466 (1.07%)
Respiratory failure	2/ 466 (0.43%)
Chronic renal disease	3/ 466 (0.64%)
Diabetes	11/ 466 (2.36%)
Hepatic chronic disease	5/ 466 (1.07%)
Autoimmune disease	42/ 466 (9.01%)
Neoplasia	20/ 466 (4.29%)
Obesity	45/ 466 (9.66%)
Allergies	36/ 466 (7.73%)
Other	47/ 466 (10.09%)

Supplementary Table S2. Side effects related to the first COVID-19 vaccine dose

	Respondents, n (%)
**Preventive treatment before first dose anti-COVID-19 vaccine (Q12)**	
Nothing	361/420 (85.95%)
Antihistamines	30/420 (7.14%)
Paracetamol	23/ 420 (5.48%)
Nonsteroidal anti-inflammatory drugs	7/ 420 (1.67%)
Yes, something else	7/420 (1.67%)
**Side effects after the first vaccine dose (Q13)**	
None	48/ 420 (11.43%)
Local cutaneous changes at injection side	29/ 420 (6.9%)
Pain at injection site	360/ 420 (85.71%)
Easy-moderate allergic reaction	3/ 420 (0.71%)
Severe allergic reaction	0/ 420 (0.00%)
Cutaneous eruptions	2/ 420 (0.48%)
Important asthenia	42/420 (10.00%)
Sleepiness	53/ 420 (12.62%)
Insomnia	7/ 420 (1.67%)
Feverish	24/ 420 (5.71%)
Fever	10/ 420 (2.38%)
Shiver	25/ 420 (5.95%)
Myalgia	39/ 420 (9.29%)
Appetit loss	7/ 420 (1.67%)
Nausea/vomiting	14/ 420 (3.33%)
Diarrhea	4/ 420 (0.95%)
Headache	45/ 420 (10.71%)
Drowsiness	24/ 420 (5.71%)
Tinnitus	2/ 420 (0.48%)
Vertigo	6/ 420 (1.43%)
Odynophagia	5/ 420 (1.19%)
Cough	5/ 420 (1/19%)
Palpitations	16/ 420 (3.81%)
Increased value of arterial tension	2/ 420 (0.48%)
Adenopathy axillar/ supraclavicular	13/ 420 (3.10%)
Something else	15/ 420 (3.57%)
**The period after the first dose in which the side effects appeared (Q14)**	
Does not apply, I did not have side effects	61/ 420 (14.52%)
Less than 24 hours	288/ 420 (68.57%)
1-2 days	62/ 420 (14.76%)
2-4 days	4/ 420 (0.95%)
4-7 days	3/ 420 (0.71%)
1-2 weeks	0/ 420 (0.00%)
Something else	2/ 420 (0.48%)
**Treatment for the anti-COVID-19 vaccine firs dose's side effects (Q15)**	
Does not apply, I did not have side effects	61/ 420 (14.52%)
Nothing, spontaneous improvement	242/ 420 (57.62%)

Antihistamines	4/ 420 (0.95%)
Paracetamol	83/ 420 (19.76%)
Nonsteroidal anti-inflammatory drugs	35/ 420 (8.33%)
Corticosteroids	1/ 420 (0.24%)
Yes, something else	15/ 420 (3.57%)

Supplementary Table S3. Diagnostic of the COVID-19 disease in the physicians' respondents to the survey

	Respondents, n (%)
**Have you been diagnosed with COVID-19? (Q24)**	
No, never	346/ 421 (82.19%)
Yes, I had one-time COVID-19	72/ 421 (17.10%)
Yes, I had two times COVID-19	1/ 421 (0.24%)
Something else	2/ 421 (0.48%)
**Moment of the COVID-19 diagnosis (Q25)**	
Before the first anti-COVID-19 vaccine	65/ 72 (90.28%)
Between the two anti-COVID-19 vaccine doses	4/ 72 (5.56%)
First two weeks after the second anti-COVID-19 vaccine dose	0/ 72 (0.00%)
First two weeks after the second anti-COVID-19 vaccine dose	0/ 72 (0.00%)
Something else	3/ 72 (4.17%)
**Time period of the COVID-19 diagnosis (Q26)**	
Mars 2020	2/ 73 (2.73%)
April 2020	3/ 73 (4.10%)
May 2020	0/ 73 (0.00%)
June 2020	2/ 73 (2.73%)
July 2020	3/ 73 (4.10%)
August 2020	3/ 73 (4.10%)
September 2020	2/ 73 (2.73%)
October 2020	13/ 73 (17.80%)
November 2020	13/ 73 (17.80%)
December 2020	20/ 73 (27.39%)
January 2021	12/ 73 (16.43%)
February 2021	0/ 73 (0.00%)
Mars 2021	0/ 73 (0.00%)
April 2021	0/ 73 (0.00%)

Supplementary Table S4. COVID-19's characteristics in the physicians' respondents to the survey

	**Respondents, n (%)**
**Disease severity for COVID-19 (Q27)**	
Asymptomatic	5/ 73 (6.85%)
Mild symptomatology	49/ 73 (67.12%)
Moderate disease severity	18/ 73 (24.66%)
Severe disease	1/ 73 (1.37%)
**COVID-19 symptomatology (Q28)**	
Important asthenia	44/ 72 (61.11%)
Fever	34/ 72 (47.22%)
Shiver	32/ 72 (44.44%)
Caught	29/ 72 (40.28%)
Dyspnea	14/ 72 (19.44%)
Anosmia/ ageusia	49/ 72 (68.06%)
Abdominal pain	5/ 72 (6.94%)
Diarrhea	16/ 72 (22.22%)
Anorexia	15/ 72 (20.83%)
Nausea/ vomiting	9/ 72 (12.50%)
Thoracic pain	17/ 72 (23.61%)
Cutaneous eruption	1 / 72 (1.39%)
Headache	38/ 72 (52.78%)
Vertigo	14/ 72 (19.44%)
Myalgias	35/ 72 (48.61%)
Something else	21/ 72 (29.17%)
**COVID-19 management (Q29)**	
Only at home	54/ 73 (73.97%)
General consultation in hospital without hospitalization	6/ 73 (8.22%)
Hospitalization, without need of oxygen therapy	10/ 73 (13.70%)
Hospitalization, with oxygen therapy without non-invasive ventilation	1/ 73 (1.37%)
Hospitalization, including non-invasive ventilation	0/ 73 (0.00%)
Hospitalization, including intubation	1/ 73 (1.37%)
Something else	1/ 73 (1.37%)
**Drugs used for COVID-19 treatment (Q30)**	
Paracetamol	64/ 73 (87.67%)
Vitamin C	60/ 73 (82.19%)
Vitamin D	60/ 73 (82.19%)
Zinc	45/ 73 (61.64%)
Famotidine	16/ 73 (21.92%)
Quercetin	15/ 73 (20.55%)
Ivermectin	0/ 73 (0.00%)
Dexamethasone	12/ 73 (16.44%)
Corticosteroids, other	0/ 73 (0.00%)
Remdesivir	2/ 73 (2.74%)
Lopinavir-Ritonavir	4/ 73 (5.48%)
Umifenovir	3/ 73 (4.11%)
Hydroxychloroquine	6/ 73 (8.22%)

Azithromycin	28/ 73 (38.36%)
Tocilizumab	2/ 73 (2.74%)
Anakinra	0/ 73 (0.00%)
Plasma	0/ 73 (0.00%)
Heparin anticoagulation	21/ 73 (28.77%)
NOAC anticoagulation	5/ 73 (6.85%)
Antiaggregant	27/ 73 (36.99%)
Something else	10/ 73 (13.7%)
